# Effects of pyrethroid resistance on the cost effectiveness of a mass distribution of long-lasting insecticidal nets: a modelling study

**DOI:** 10.1186/1475-2875-12-77

**Published:** 2013-02-25

**Authors:** Olivier JT Briët, Melissa A Penny, Diggory Hardy, Taiwo S Awolola, Wim Van Bortel, Vincent Corbel, Roch K Dabiré, Josiane Etang, Benjamin G Koudou, Patrick K Tungu, Nakul Chitnis

**Affiliations:** 1 Department of Epidemiology and Public Health, Swiss Tropical and Public Health Institute, Basel, Switzerland; 2 University of Basel, Basel, Switzerland; 3 Molecular Entomology and Vector Control Research Laboratory, Nigerian Institute of Medical Research, PMB 2013, Yaba Lagos, Nigeria; 4 Department Parasitology - Entomology, Institute of Tropical Medicine, Nationalestraat 155, B-2000, Antwerpen, Belgium; 5 Institut de Recherche pour le Développement (IRD), Maladies Infectieuses et Vecteurs, Ecologie, Génétique, Evolution et Contrôle (IRD 224-CNRS 5290 UM1-UM2), Montpellier Cedex 5, France; 6 Department of Entomology, Faculty of Agriculture, Kasetsart University, 10900, Bangkok, Thailand; 7 Institut de Recherche en Sciences de la Santé (IRSS)/Centre Muraz (CM), 01 BP 390, Bobo-Dioulasso, Burkina Faso; 8 Organisation de Coordination pour la lutte contre les Endémies en Afrique Centrale (OCEAC), BP. 288, Yaoundé, Cameroon; 9 Faculty of Medicine and Pharmaceutical Sciences, University of Douala, P.O. Box 2701, Douala, Cameroon; 10 Département Environnement et Santé, Centre Suisse de Recherches Scientifiques, 01 01 BP 1303, Abidjan, Côte d’Ivoire; 11 UFR Sciences de la Nature, Université d’Abobo-Adjamé, 02 02 BP 801, Abidjan, Côte d’Ivoire; 12 Centre for Neglected Tropical Diseases, Liverpool School of Tropical Medicine, L3 5QA, Liverpool, UK; 13 Amani Medical Research Centre, National Institute for Medical Research, P.O. Box 81, Muheza, Tanzania

**Keywords:** Pyrethroid, Insecticide, Resistance, LLIN, ITN, Modelling, Piperonyl butoxide

## Abstract

**Background:**

The effectiveness of insecticide-treated nets in preventing malaria is threatened by developing resistance against pyrethroids. Little is known about how strongly this affects the effectiveness of vector control programmes.

**Methods:**

Data from experimental hut studies on the effects of long-lasting, insecticidal nets (LLINs) on nine anopheline mosquito populations, with varying levels of mortality in World Health Organization susceptibility tests, were used to parameterize malaria models. Both simple static models predicting population-level insecticidal effectiveness and protection against blood feeding, and complex dynamic epidemiological models, where LLINs decayed over time, were used. The epidemiological models, implemented in OpenMalaria, were employed to study the impact of a single mass distribution of LLINs on malaria, both in terms of episodes prevented during the effective lifetime of the batch of LLINs, and in terms of net health benefits (NHB) expressed in disability-adjusted life years (DALYs) averted during that period, depending on net type (standard pyrethroid-only LLIN or pyrethroid-piperonyl butoxide combination LLIN), resistance status, coverage and pre-intervention transmission level.

**Results:**

There were strong positive correlations between insecticide susceptibility status and predicted population level insecticidal effectiveness of and protection against blood feeding by LLIN intervention programmes. With the most resistant mosquito population, the LLIN mass distribution averted up to about 40% fewer episodes and DALYs during the effective lifetime of the batch than with fully susceptible populations. However, cost effectiveness of LLINs was more sensitive to the pre-intervention transmission level and coverage than to susceptibility status. For four out of the six *Anopheles gambiae* sensu lato populations where direct comparisons between standard LLINs and combination LLINs were possible, combination nets were more cost effective, despite being more expensive. With one resistant population, both net types were equally effective, and with one of the two susceptible populations, standard LLINs were more cost effective.

**Conclusion:**

Despite being less effective when compared to areas with susceptible mosquito populations, standard and combination LLINs are likely to (still) be cost effective against malaria even in areas with strong pyrethroid resistance. Combination nets are likely to be more cost effective than standard nets in areas with resistant mosquito populations.

## Background

Malaria control is based on both preventing transmission and promptly and effectively treating infection. Many countries have made significant progress in preventing malaria by focusing largely on vector control through long-lasting insecticidal nets (LLINs) and indoor residual spraying (IRS) of insecticides [1]. In areas with insecticide-susceptible mosquito populations, the insecticide on LLINs mitigates the loss of personal protection if the net becomes holed. Furthermore, the nets have a community effect by reducing the longevity of malaria vector mosquitoes [2]. Over the last decade, many countries have significantly increased LLIN coverage with great impact: it is estimated that between 2000 and 2010, LLINs saved over 908,000 lives, with three quarters of those deaths having been prevented since 2006 [3]. However, both IRS and LLINs face the development of physiological resistance (against insecticide) and ‘behavioural resistance’ in mosquitoes, which can reduce effectiveness of these interventions and possibly reverse the gains made in reducing malaria morbidity [4]. Although such resistance may be inevitable with successful control programmes, new strategies need to be developed to mitigate development and spread of insecticide resistance and to preserve the efficacy of currently available insecticides and the effectiveness of malaria control interventions.

The problem of physiological resistance against insecticides is more acute for LLINs than for IRS, as LLINs rely solely on pyrethroids, whereas IRS can be done with several classes of insecticides. There is evidence of increasing levels of pyrethroid resistance [5] and corresponding decreases in the effectiveness of malaria control programmes that rely on pyrethroid-based interventions [6,7]. Although LLINs containing non-pyrethroid classes of insecticides are under development, no such net currently exists. However, to mitigate the effects of pyrethroid resistance, nets using a combination of a pyrethroid and a synergist (piperonyl butoxide) have been developed.

The aim of this study is to assess the sensitivity of the effectiveness of a mass distributed batch of LLINs to insecticide resistance, both in terms of episodes averted during the effective life time of the batch, and in terms of net health benefits (NHB) expressed in disability adjusted life years (DALYs) averted.

## Methods

### Field data

Recently, the effects of a deltamethrin and piperonyl butoxide (PBO) combination mosquito net, PermaNet 3.0 (P3), on blood feeding and mortality were studied in experimental hut studies [8-12] in seven locations in Africa^a^ on eight different *Anopheles gambiae* sensu lato populations (populations 1–8 in Table 1). In another location in Vietnam, it was tested on *Anopheles epiroticus*[13] (population 9 in Table 1). The studies in these locations were of similar design, followed similar protocols, and assessed blood feeding and mortality of P3 *versus* standard LLINs containing a single insecticide. P3 has a polyethylene roof incorporated with deltamethrin (at a target dose of 4 g/kg +/− 25%) and PBO at a target dose of 25 g/kg +/− 25%. The 75-denier polyester sides have a knitted 70 cm border region, coated with a wash-resistant binder and deltamethrin at a target dose of 2.8 g/kg +/− 25%. The deltamethrin target dose for P2 nets is lower, at 1.8 g/kg and 1.4 g/kg for 75-denier and 100-denier nets, respectively, +/− 25%.

**Table 1:** Data sources

**Population**		**Start**	**Lat.**	**Lon.**	**AG (%)**	**Deltamethrin 0.05%**		**Permethrin 0.75%**		**Ref.**
						**† (%)**	**n**	**† (%)**	**n**	
1	Pitoa	Jul-08	9.38	13.53	5	70.0	100			[8]
2	Kou	Sep-07	11.4	4.4	100	23.0	100			[8]
3	Akron	Oct-08	6.47	2.63	100	1.3^d^		23.0^e^	80	[9,29]
4	Malanville	Jul-08	11.87	3.38	95	85.0	100	99	101	[8]
5	Zeneti	Jul-08	−5.22	38.65	100^a^	100.0	50			[10,30]
6	New Bussa AG	Jul-10	9.88	4.52	100^b^	79.5	132	75.7	140	[12]
7	New Bussa AA	Jul-10	9.88	4.52	0^b^	100.0	118	100.0	102	[12]
8	Yaokoffikro	Apr-09	7.18	−5.02	100^c^	10.6	90	43.9	90	[11,31]
9	Vand Duc A	Sep-08	9.18	105.3	AE	75	100			[13]

Legend:

*Lat*.: latitude; *Lon*.: longitude; *AG*: *Anopheles gambiae* sensu stricto, the complement is *Anopheles arabiensis*; *AA*: *Anopheles arabiensis*; *Ref*.: Reference; †: mortality; *n*: (approximate) number of mosquitoes tested for one hour in WHO susceptibility (tube) tests with insecticide on filter papers [14]; *AE*: *Anopheles epiroticus*; ^a^Based on Kitau and colleagues [30]; ^b^During the study, the *An*. *gambiae* s.l. population was composed of 62% *An*. *gambiae* s.s. and 38% *An*. *arabiensis*; ^c^Presumably, based on Chandre and colleagues [31]; ^d^Based on linear extrapolation of the relationship of logit transformed mortality with deltamethrin and permethrin, using data from ‘New Bussa AG’ and ‘Yakoffikro’; ^e^For the WHO susceptibility test, mosquitoes were collected December 2006–January 2007.

For all these mosquito populations, except ‘Akron’ (population 3), results were available from World Health Organization (WHO) susceptibility test [14] assays with 0.05% deltamethrin (Table 1) conducted at the time of the experimental hut studies. However, for ‘Akron’, as well as for ‘New Bussa AA’, ‘New Bussa AG’ and ‘Yaokoffikro’, data on susceptibility to 0.75% permethrin was available, and this permitted estimating of the mortality of ‘Akron’ if exposed to 0.05% deltamethrin in WHO tests, assuming a linear^b^ relationship between logit transformed mortality proportions of permethrin and deltamethrin. The nine mosquito populations (populations 1–9 in Table 1) varied in their susceptibility to deltamethrin over a wide range from susceptible to resistant.

Summarised experimental hut data on the numbers of unfed alive (UA), unfed dead (UD), fed alive (FA) and fed dead (FD) female mosquitoes for selected study arms are shown in Additional file 1. Some of these data were not previously published in as much detail. For populations 6 and 7, the data are for the first six weeks of data collection, whereas the reference available for this study [12] summarises the total of 12 weeks of data collection, and gives detailed results for ‘New Bussa AG’ (population 6 in Table 1) and a *Culex quinquefasciatus* population not used here, but not for ‘New Bussa AA’ (population 7 in Table 1). For population 8, the study [11] deviates from the standard protocol, due to ethical concerns, in that intact rather than purposely holed nets were used. For populations 1–7 and 9, studies [8-10,12,13] do not have study arms with intact nets. To assess the cost effectiveness of a mass distributed batch of LLINs, in the simulations, nets were presumed to be delivered intact and develop holes over time due to wear and tear. Because the studies test either artificially holed or intact nets but not both, additional data from experimental hut studies [15,16] elsewhere (populations 10 and 11 in Additional files 1 and 2) were used to estimate effects, depending on the physical state in terms of holed area in the fabric surface.

For populations 1–9, and 10, studies do not have controls with unprotected individuals (i.e., a person without a net). For population 10, only summarised data were available and numbers for the four mosquito categories (FA, FD, UD, UA) were estimated assuming that the number of FD was the average of the number of fed mosquitoes multiplied by the proportion of FD out of fed mosquitoes and the number of dead mosquitoes multiplied by the proportion of FD out of dead mosquitoes in the corresponding arm of the study on population 11.

For the studies on populations 1–4 and 6–7, insecticide content after completion of the experimental hut studies in side panels in g/kg was converted into mg/m^2^ units. The conversion factor was 55/1.8 = 30.6 for 75 denier nets was and 55/1.4 = 39.3^c^ for 100 denier nets and were derived from WHO specifications [17]. For the study on populations 5 and 9, this was already in mg/m^2^. For the study on population 8, no insecticide content data were available. Therefore, these were estimated as follows: for the unwashed nets, the insecticide content was estimated as the mean target dose (P2: 1.8 g/kg; P3: 2.8 g/kg), multiplied by the average percentage retention after completion of the experimental hut essays, found in the studies on populations 1–5 (P2: 91.7%; P3: 95.7%); for the 20-times washed nets, the target dose was multiplied by the average percentage retention found in the studies on populations 1–5 after washing and completion of the experimental hut studies (P2: 27.4%; P3: 24.0%).

Parameter values for the effects of LLINs on mosquito populations needed in the mathematical models were estimated according to the results from these experimental hut studies (see Additional files 1 and 2). These parameter values were used both for analysing the relationship between insecticide resistance status and the population level insecticidal effectiveness and protection against feeding [18] of new and decayed LLINs, and for simulation studies on the malaria epidemiological effectiveness of LLINs.

### Simulations

**Figure 1:**
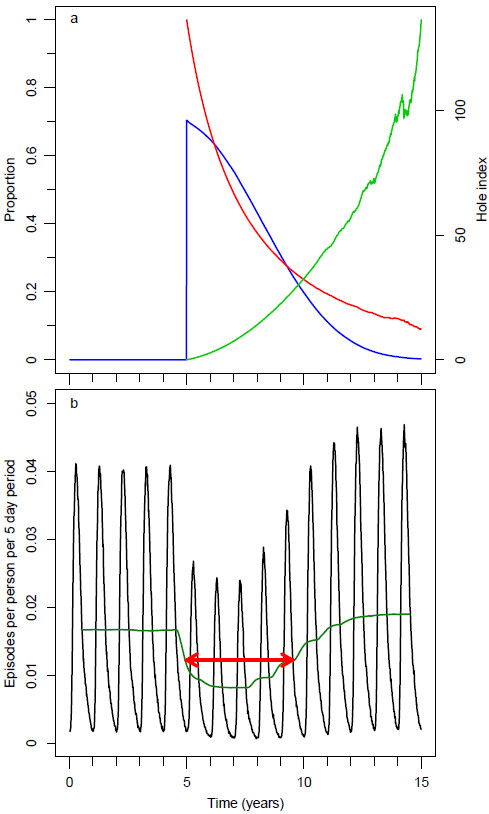
**Central scenario simulation with base model. a**) The blue line (on left vertical axis) represents the proportion of the population covered, the red line (on left vertical axis) represents the mean insecticide in the remaining LLINs as a proportion of its initial value. The light green line (on the right vertical axis) represents the mean hole index in the remaining LLINs. **b**) The black line represents the number of episodes per person per five-day period. The dark green line represents the one-year moving average of the number of episodes per person per five-day period. The red arrow indicates the approximate length of the effective lifetime of the LLIN distribution.

Simulations were run using the OpenMalaria modelling platform [19-21], which combines stochastic individual-based models for *Plasmodium falciparum* malaria in humans with a deterministic model for malaria in mosquitoes [20,22]. The experiment was based on the ‘central scenario’ used by Briët and colleagues [23], who explain the parameterization in detail. Only in sections describing the vector population, LLINs and the effects of LLINs on vectors, parameter values were different, depending on the experimental hut data. Since OpenMalaria currently does not support the modelling of *Plasmodium vivax*, present along with *P*. *falciparum* in Van Duc A [24], malaria in this setting was not simulated. The ‘central scenario’ used in this study is discussed in detail in the Additional file 2. Briefly, LLINs were distributed to the simulated population in a single mass distribution, after which they diminished in number following an attrition curve with a half life of four years, and decayed chemically (loss of insecticide) and physically (formation of holes in the fabric). In the experiment, 14 model variants [25] were run at a range of pre-intervention transmission levels for non-intervention and intervention scenarios. In intervention scenarios, the effects of LLINs (P2 or P3) were varied, depending on the mosquito population. Also, coverage and pre-intervention entomological inoculation rate (EIR) was varied. For each scenario, 10 simulations were run, each with a unique random seed. Figure 1 illustrates a simulation run with a central scenario.

From each scenario output, based on the number of uncomplicated episodes, severe episodes, sequelae and deaths depending on age, DALYs and health system costs (HSC) were calculated for each whole year during the simulation run (see Additional file 2). For intervention scenarios, the ‘effective lifetime’ (the period of the epidemiological effect from the year of distribution until the year where the effect had waned to just over half of the maximum impact achieved in terms of uncomplicated and severe episodes averted) as compared to non-intervention scenarios, was determined. This period is illustrated by the red arrow in Figure 1b. For each scenario and corresponding non-intervention scenario, the outcome variables during this period were averaged. This allowed calculation of the effectiveness of a mass distributed batch of LLINs in terms of episodes averted during the effective lifetime, and cost effectiveness in terms of NHB. The NHBs were calculated using a value of 1 DALY = 235.28 (2012) USD, as described in Additional file 3.

## Results and discussion

### Insecticide resistance status and LLIN effects in static models

**Figure 2:**
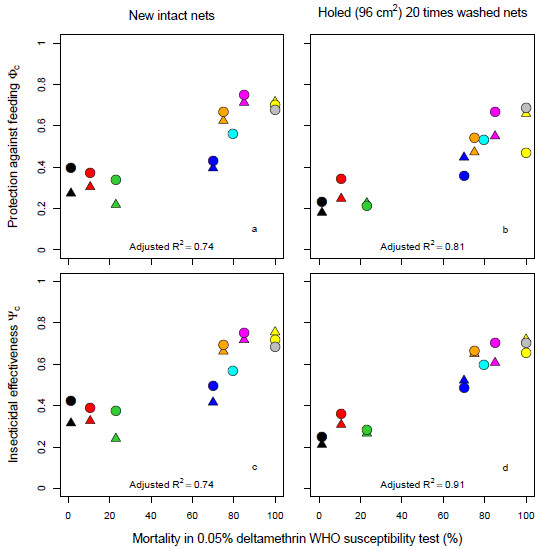
**Population level effectiveness of new and aged nets, depending on mortality in WHO susceptibility tests.** First column of panels (**a** &**c**) for intact new nets, second column of panels (**b** &**d**) for 20 times washed nets with a holed area of 96 cm^2^. The first row of panels (**a**, **b**) shows population level protection against feeding of mosquitoes if 70% of the human population uses a net. The second row of panels (**c**, **d**) shows the corresponding population level insecticidal effectiveness, as defined by Briët and colleagues [18]. Circles and triangles represent estimated values for PermaNet 3.0 and PermaNet 2.0, respectively. Colours represent mosquito populations 1–9 in Table 1, with black: ‘Akron’, red: ‘Yaokoffikro’, lime green: ‘Kou’, orange: ‘Van Duc A’, dark blue: ‘Pitoa’, cyan: ‘New Bussa AG’, magenta: ‘Malanville’, yellow: ‘Zeneti’, and grey: ‘New Bussa AA’. Adjusted R^2^ values were calculated using linear regression on logit transformed proportions.

Figure 2 shows the relationship between the (estimated) mortality of adult female mosquitoes from eight *An*. *gambiae* s.l. populations (populations 1–8 in Table 1) and one *An*. *epiroticus* (population 9) exposed to 0.05% deltamethrin in WHO susceptibility tests and calculated population level protection against feeding and insecticidal effectiveness [18] if 70% of the population uses nets, using parameter value estimates based on experimental hut data (Additional files 1 and 2).

The strong correlation between the population level direct protection against feeding and the mortality in WHO susceptibility assays is striking: whereas WHO susceptibility assays cannot assess behavioural resistance, results derived from experimental hut assays are affected by physiological or biochemical resistance, deterrence and dissuasion from attacking. These last two effects, resulting in avoidance of contact with the insecticide, could be seen as a form of behavioural resistance, even though it is possibly effective in reducing host-vector contact. Note that these relationships change as LLINs decay (first column of panels *versus* second column of panels), but that this is unimportant relative to the differences observed among populations.

Although the effectiveness of an intervention programme against malaria transmission depends both on protection against bites and the insecticidal effectiveness, more complex models, such as those implemented in OpenMalaria, are required to estimate impacts on transmission and morbidity. Nevertheless, these relatively simple measures provide some predictions against which the predictions of the more complex models can be checked. Since Figure 2 shows strong correlation with susceptibility status and both protection against feeding and insecticidal effectiveness, such a result can also be expected for transmission predictions by the more complex models. For new intact nets, where comparisons between P2 and P3 are possible, it appears that P3 is more effective than P2 in most situations, except for the fully susceptible population ‘Zeneti’: both protection against feeding and insecticidal effectiveness were different for the two LLIN types (Wilcoxon signed rank test, alpha = 0.05). For decayed nets, protection against feeding by P3 was higher than P2 in four out of seven populations, and insecticidal effectiveness of P3 was higher than P2 in five out of seven populations, but these differences were not significant.

### LLIN effectiveness depending on transmission level

**Figure 3:**
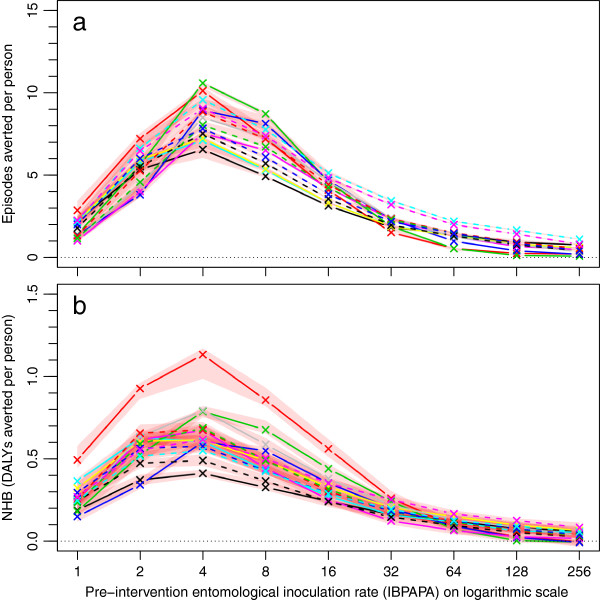
**LLIN effectiveness depending on the pre-intervention entomological inoculation rate.** Each line represents the median number of **a**) episodes averted or **b**) net health benefits (NHB), which are expressed in disability adjusted life years (DALYs) averted per person, of 10 simulation runs (each with unique random seed) with PermaNet 3.0 bed nets distributed to 70% of the people (population size = 100,000) during the effective lifetime of a mass distribution, as compared to matching non intervention scenarios, with a susceptible mosquito population (‘Zeneti’), of which pre-intervention, 75% was determined to always search for hosts indoors during times that prospective LLIN users would be under their nets, and 25% always searched for hosts at other times. The red semi transparent polygons represent the range of the 10 runs. Per panel, there are 14 lines (and 14 red polygons), each representing a malaria model variant. Model variants [25]: R0000 = solid black lines and crosses; R0063 = solid red lines and crosses; R0065 = solid lime green lines and crosses; R0068 = solid blue lines and crosses; R0111 = solid cyan lines and crosses; R0115 = solid magenta lines and crosses; R0121 = solid yellow lines and crosses; R0125 = solid grey lines and crosses; R0131 = dashed black lines and crosses; R0132 = dashed red lines and crosses; R0133 = dashed lime green lines and crosses; R0670 = dashed blue lines and crosses; R0674 = dashed cyan lines and crosses; R0678 = dashed magenta lines and crosses. The horizontal axis shows the pre-intervention entomological inoculation rate expressed in infectious bites per adult per annum (IBPAPA). Horizontal dotted lines are at zero episodes or DALYs averted.

Figure 3 shows how the LLIN (P3) effectiveness varied depending on the pre-intervention EIR with an insecticide-susceptible mosquito population (‘Zeneti’) in terms of episodes averted (Figure 3a) and NHB (Figure 3b). Figure 3a and b look similar, apart from different scales on the vertical axes. This is despite the fact that the majority of episodes account for only a small proportion of the DALYs on which NHB are based^d^. In non-intervention scenarios, depending on model variant and transmission level, episodes that do not result in sequelae or death account for 99.4–99.8% of all episodes, yet account for only 1.6–5.4% of DALYs. The ranges of results per model variant (red polygons) are somewhat wider for NHB than for episodes, because deaths, the most important component of NHB (see Additional file 3), are relatively stochastic events. In Figure 3a, model variants appear to behave similarly, with two variants, R0674 (uncorrelated heterogeneities in access to treatment and susceptibility to co-morbidity) and R0678 (heterogeneity in access to treatment) [25], showing somewhat higher values at the higher pre-intervention EIR levels. In Figure 3b, the model variant R0063 (availability of humans to mosquitoes varies between hosts [25]) showed a higher number of DALYs averted than the other 13 model variants at pre-intervention EIRs lower than 32 IBPAPA. The optimum pre-intervention EIR was around four infectious bites per person per annum (IBPAPA) for most entomological settings, where the LLIN distribution averts six to 11 episodes or NHB are 0.4–1.1 DALYs per person.

### Insecticide resistance status and LLIN effectiveness depending on transmission level

**Figure 4:**
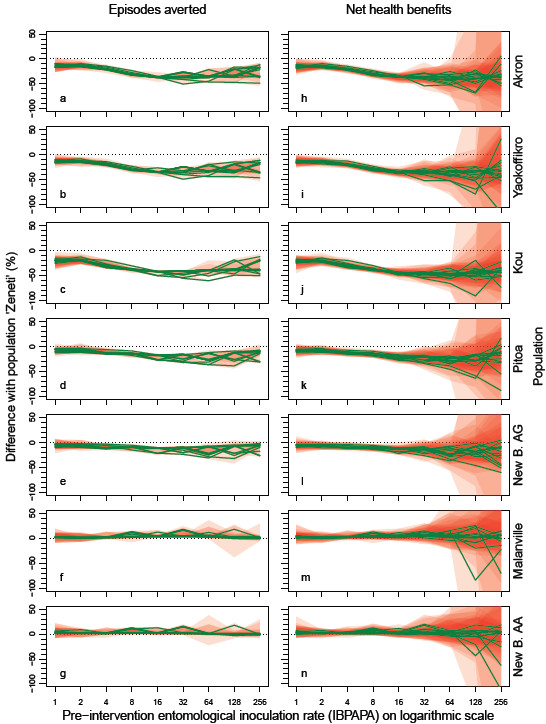
**Effectiveness of a mass distribution of PermaNet 3.0 bed nets, depending on insecticide resistance status, compared to a susceptible population.** Each green line represents the median difference between a population and population ‘Zeneti’, as percentage of ‘Zeneti’, in episodes averted (panels **a**–**g**) and net health benefits (panels **h**–**n**) of 10 simulation runs (each with unique random seed) with nets distributed to 70% of the people (population size = 100,000) during the effective lifetime of a mass distribution, as compared to matching non intervention scenarios, assuming that prior to intervention, 75% of the mosquito population was determined to always search hosts during times when prospective LLIN users would be protected by their nets, and the remainder (25%) always searched hosts during other times. The red semi-transparent polygons represent the range of the 10 runs. Per panel, there are 14 green lines (and 14 red polygons), each representing a malaria model variant. ‘New Bussa’ is abbreviated as ‘New B.’ Populations are shown from top to bottom in order of mortality in 0.05% deltamethrin WHO susceptibility tests, with the least mortality at the top. The horizontal axis shows the pre-intervention entomological inoculation rate expressed in infectious bites per adult per annum (IBPAPA). Horizontal dotted lines are at zero difference.

The effect of insecticide resistance status on LLIN effectiveness is illustrated by Figure 4 for P3, by comparing the number of episodes averted (Figure 4a–g) and the NHB (Figure 4h–n) for each mosquito population with population ‘Zeneti’, which was one of the two populations with full susceptibility (100% mortality in 0.05% deltamethrin WHO susceptibility tests). For all populations except the two most susceptible (‘New Bussa AA’ and ‘Malanville’ with 100% and 85% mortality in 0.05% deltamethrin WHO susceptibility tests, respectively) LLINs were less effective than with population ‘Zeneti’. With population ‘Akron’, ranked as the most resistant to deltamethrin, LLINs were up to about 40% less effective than with ‘Zeneti’, but this varied with pre-intervention EIR. The relationships were similar for P2 (See Additional file 4).

### LLIN effectiveness depending on LLIN type

**Figure 5:**
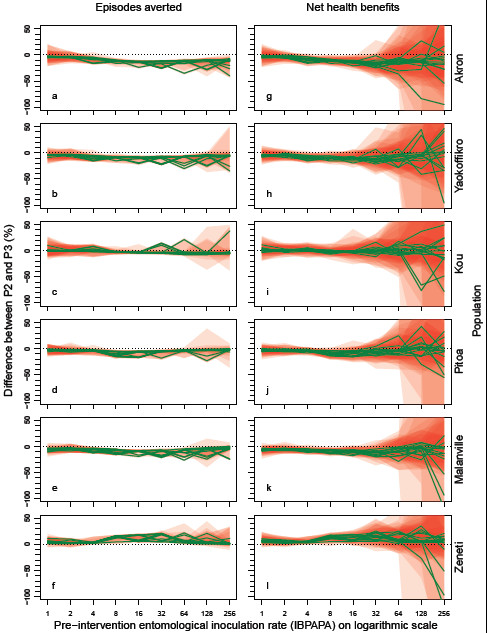
**Difference in effectiveness of PermaNet 2.0 and 3.0 bed nets, depending on the entomological situation.** Each green line represents the median difference in malaria episodes averted (Panels **a**–**f**) and net health benefits (Panels **g**–**l**) between mass distributions of PermaNet 2.0 (P2) and PermaNet 3.0 (P3) bed nets, as percentage of effectiveness of P3, of 10 simulation runs (each with unique random seed) with nets distributed to 70% of the people (population size = 100,000) during the effective lifetime of a mass distribution, as compared to matching non intervention scenarios. Prior to intervention, 75% of the mosquito-host interaction occurred during times when people were indoors and asleep, and host-searching behaviour was assumed fully determined (a mosquito will display the same behaviour as in the first gonotrophic cycle, each following gonotrophic cycle). A positive difference indicates that P2 prevents more episodes than P3. Per panel, there are 14 green lines (and 14 red polygons), each representing a malaria model variant. Populations are shown from top to bottom in order of mortality in 0.05% deltamethrin WHO susceptibility tests, with the least mortality at the top. The horizontal axis shows the pre-intervention entomological inoculation rate expressed in infectious bites per adult per annum (IBPAPA). Horizontal dotted lines are at zero difference.

Figure 5 shows the difference in expected effectiveness if P2 was used instead of P3, depending on insecticide susceptibility in the population and pre-intervention EIR.

The difference between the two LLIN types, up to about 15% in the most resistant population, was generally small, compared to the effect of insecticide susceptibility on LLIN effectiveness (Figure 4). The strongest differences were found at transmission levels between eight and 32 IBPAPA.

With three out of four resistant populations (‘Akron’, ‘Yaokoffikro’ and ‘Pitoa’), P3 was more effective, whereas with population ‘Kou’ there was no apparent difference. With one fully susceptible population (‘Malanville’), P3 was more effective, whereas with the fully susceptible population (‘Zeneti’), P2 was more effective.

### Sensitivity analysis

**Figure 6:**
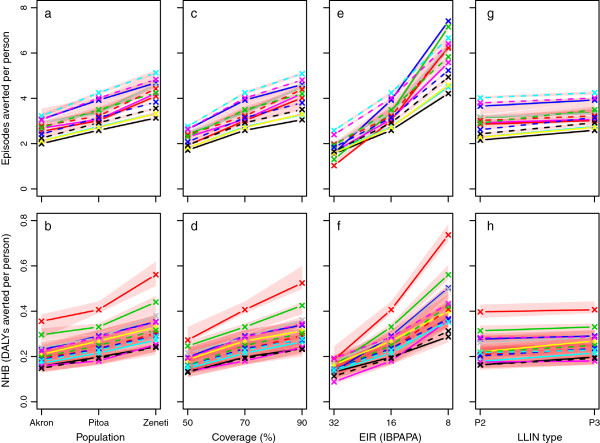
**Mass LLIN distribution’s effectiveness depending on insecticide susceptibility, coverage, transmission level and LLIN type.** Each line represents the median number of episodes averted per person (Panels **a**, **c, e** &**g**) or net health benefits (NHB), which are expressed in disability adjusted life years (DALYs) averted per person (Panels **b**, **d, f** &**h**) of 10 simulation runs (each with unique random seed) during the mass distribution’s effective lifetime, as compared to matching non intervention scenarios, with population size 100,000. Model variant lines and red area are as in Figure 3. In the first column of panels (Panels **a** &**b**), the susceptibility to pyrethroids is varied from the most resistant population, population ‘Akron’ with estimated 1.3% mortality in 0.05% deltamethrin WHO susceptibility tests, to a less resistant population, population ‘Pitoa’ (70% mortality), to a fully susceptible population, population ‘Zeneti’ (100% mortality). The pre-intervention entomological inoculation rate (EIR) was 16 infectious bites per adult per annum (IBPAPA), and the coverage of PermaNet 3.0 (P3) LLINs was initially 70%. In the second column (Panels **c** &**d**), the initial coverage of P3 LLINs was varied between 50 and 90%. The mosquito population was ‘Pitoa’ and prior to intervention, the EIR was 16 IBPAPA. In the fourth column (Panels **e** &**f**), the pre-intervention EIR was varied between 32 and 8 IBPAPA (note the decreasing scale), with 70% initial P3 LLIN coverage and susceptibility of mosquito population ‘Pitoa’. In the fifth column (Panels **g** &**h**), P3 nets are compared to P2 at an EIR of 16 IBPAPA with 70% initial LLIN coverage and susceptibility of mosquito population ‘Pitoa’. Note that for each model variant, the middle value displayed (right hand value for the fifth column of panels) is the same for all panels in a row of panels.

Figure 6 illustrates the sensitivity of the effectiveness of a mass LLIN distribution to insecticide susceptibility, initial LLIN coverage, pre-intervention EIR and LLIN type around a central scenario with P3 LLINs. As observed in a study with only the effective lifetime of a net distribution as outcome [23], the pre-intervention EIR was extremely important. Even if varied over a small part over its potential range, the pre-intervention EIR had more impact on the effectiveness of an LLIN distribution than did deltamethrin susceptibility of mosquito populations, for which mortality in 0.05% deltamethrin WHO susceptibility test varies from 1.3% to 100%. The sensitivity to the initial coverage (which was varied between 50 and 90%) was slightly more important than the susceptibility status. The sensitivity to the LLIN type was relatively weak, but still, in this central scenario setting, upgrading from P2 to P3 was associated with an increase in NHB, despite the higher costs of P3. It should be noted that the sensitivity to LLIN type was strongly dependent on the mosquito population. For example, with the more resistant populations ‘Akron’ and ‘Yaokoffikro’, sensitivity to LLIN type was somewhat higher (Figure 5).

## Conclusions

Simple static models that predict population level insecticidal effectiveness and protection against blood feeding, and complex dynamic epidemiological models agreed that insecticide susceptibility, as measured by mortality in WHO susceptibility tests, is strongly correlated with LLIN effectiveness against malaria. An earlier modelling study by Killeen and colleagues [26] on the effectiveness of P3 and P2 LLINs in reducing malaria transmission provides a ‘snapshot’ of how transmission might be affected with nets in a certain physical and chemical state, when the mosquito population structure is in an equilibrium state. In the present simulation experiment, the LLINs were followed from a new and intact state along the path of attrition and physical and chemical decay, while considering the interacting dynamics of malaria in humans and mosquitoes. Nevertheless, both studies agree that, taking community level effects into account, both P3 and P2 nets are (still) effective in suppressing malaria in areas with resistant mosquito populations. Despite being more expensive, P3 was more cost effective than P2 against three out of four resistant populations. This could be due to the PBO present in the roofs of P3 nets, but effects of the higher deltamethrin content of P3 nets cannot be excluded [8]. However, for the areas where mosquitoes are (still) fully susceptible, results were inconclusive, with P3 being more cost effective in one area and P2 being more cost effective in another. Despite resistance being an important factor in reducing the effectiveness of LLINs, both when measured in terms of averted episodes and in terms of NHB, coverage level and especially the pre-intervention transmission level appear still more important. In settings with moderate pre-intervention transmission, a mass distributed batch of LLINs was cost effective against malaria even in the presence of strong physiological or biochemical resistance. However, at pre-intervention transmission levels above 128 IBPAPA, a minority of variants of the model ensemble showed (slightly) negative net health benefits, even with fully susceptible mosquitoes. Moreover, it should be noted that in most malaria endemic countries, malaria control will not be limited to a single mass distribution of LLINs, and cost-effectiveness may alter with prolonged LLIN use.

Nevertheless, standard and combination LLINs are likely to be cost effective against malaria even in areas with strong pyrethroid resistance, and LLINs remain an effective transmission control paradigm in the fight against malaria.

## Endnotes

^a^The earlier comparative modelling study by Killeen and colleagues [26] used data for four populations (Pitoa, Kou, Akron, and Van Duc A, there named ‘Cameroon’, ‘Burkina Faso’, ‘Benin’, and ‘Vietnam’, respectively) of the nine used here, to study the effect of holed P3 and P2 nets on human exposure to infectious bites.

^b^Deltamethrin and permethrin cross resistance is likely mechanism dependent and the assumption of a linear relationship could be biased, since in population ‘Akron’, esterase, kdr and oxidase mechanisms were reported, whereas in populations ‘New Bussa AG’ and ‘Yakoffikro’ only kdr and oxidase were reported. It seems reasonable to assume that the mortality of ‘Akron’ to deltamethrin would be less than that of population ‘Yaokoffikro’, based on lower mortality of *An*. *gambiae* s.s. in Asecna and Ladji [27] (neighbouring Akron) than in Yaokoffikro [11,28].

^c^Polyester, 75 denier netting weighs approximately 30 g/m^2^ +/− 10%, and 100 denier weighs approximately 40 g/m^2^ +/− 10%.

^d^NHBs are only slightly lower than DALYs averted during the effective lifetime of a mass distributed batch of LLINs, because the health systems cost of a net distribution are relatively low compared to DALYs averted if expressed in terms of DALYs (see Additional file 3).

## Competing interests

The experimental hut field studies testing P3 used to parameterize the model, and the modelling study itself, were funded by Vestergaard-Frandsen. The study in Zeneti [8] was conducted through the WHO Pesticide Evaluation Scheme (WHOPES). The study in Van Duc A [13] was supported by the Belgian Directorate-General for Development Cooperation and WHOPES, with Vestergaard-Frandsen providing the nets. Dr Kim, Dr Pates Jamet, Dr Knox and Dr Bwambok of Vestergaard Frandsen commented on the manuscript. The findings described in this manuscript are those of the authors and do not necessarily reflect views of Vestergaard Frandsen.

## Authors’ contributions

OJTB designed the experiments, analysed results and drafted the manuscript. MAP provided mathematical support. DH implemented modifications to the code in OpenMalaria. TSA, WVB, VC, RKD, JE, BGK and PKT conducted the experimental hut studies. NC conceived of the study and participated in the design. All authors read and approved the final manuscript.

## Supplementary Material

Additional file 1 Summarized data from experimental hut studies. Click here for file

Additional file 2 Experiment parameterization and parameter values. Click here for file

Additional file 3 Health economics. Click here for file

Additional file 4 **Effectiveness of a mass distribution of PermaNet 2.0 bed nets depending on insecticide resistance status, compared to a susceptible population; Legend: See Figure **4**.** Click here for file
