# Supplementary material for: Effects of pyrethroid resistance on the cost effectiveness of a mass distribution of long-lasting insecticidal nets: a modelling study
Source: Malar J. 2013 Feb 25;12:77. doi: 10.1186/1475-2875-12-77 (PMC3598792; doi:10.1186/1475-2875-12-77)
Supplement: Additional file 1 — Summarized data from experimental hut studies. [file 1475-2875-12-77-S1.pdf]

Additional file 1. Summarized data from experimental hut studies

Population	Arm	UA	FA	FD	UD	Total	%F	%D	*p* (mg/m^2)
1 Pitoa	C,h96	154	195	14	38	401	52.1	13.0	0.00
	P2x0,h96	11	7	9	78	105	15.2	82.9	76.69
	P3x0,h96	2	7	34	103	146	28.1	93.8	76.39
	P2x20,h96	99	36	27	148	310	20.3	56.5	12.53
	P3x20,h96	23	13	27	100	163	24.5	77.9	10.08
2 Kou	C,h96	185	679	8	36	908	75.7	4.8	0.00
	P2x0,h96	90	93	23	123	329	35.3	44.4	51.86
	P3x0,h96	53	48	48	314	463	20.7	78.2	115.50
	P2x20,h96	199	351	31	207	788	48.5	30.2	10.61
	P3x20,h96	175	192	71	286	724	36.3	49.3	35.36
3 Akron	C,h96	43	113	8	21	185	65.4	15.7	0.00
	P2x0,h96	14	50	14	36	114	56.1	43.9	60.00
	P3x0,h96	12	49	12	55	128	47.7	52.3	96.00
	P2x20,h96	50	73	8	43	174	46.6	29.3	8.80
	P3x20,h96	29	78	7	41	155	54.8	31.0	8.40
4 Malanville	C,h96	169	104	3	9	285	37.5	4.2	0.00
	P2x0,h96	21	6	3	213	243	3.7	88.9	53.17
	P3x0,h96	7	0	1	206	214	0.5	96.7	70.89
	P2x20,h96	51	6	6	132	195	6.2	70.8	30.25
	P3x20,h96	49	14	13	134	210	12.9	70.0	16.19
5 Zeneti	C,h96	442	173	29	79	723	27.9	14.9	0.00
	P2x0,h96	20	6	53	495	574	10.3	95.5	61.00
	P3x0,h96	18	0	11	396	425	2.6	95.8	100.00
	P2x20,h96	62	14	40	470	586	9.2	87.0	25.00
	P3x20,h96	23	4	54	477	558	10.4	95.2	48.00
6 New Bussa AG	C,h96	79	112	3	6	200	57.5	4.5	0.00
	P3x0,h96	39	0	4	104	147	2.7	73.5	85.00
	P3x20,h96	57	1	6	88	152	4.6	61.8	34.00
7 New Bussa AA	C,h96	67	58	2	7	134	44.8	6.7	0.00
	P3x0,h96	4	0	1	80	85	1.2	95.3	85.00
	P3x20,h96	9	0	2	85	96	2.1	90.6	34.00
8 Yaokoffikro	C,h0	674	65	0	52	791	8.2	6.6	0.00
	P2x0,h0	177	11	5	97	290	5.5	35.2	*60.54
	P3x0,h0	166	19	7	166	358	7.3	48.3	*91.76
	P2x20,h0	139	18	8	99	264	9.8	40.5	*17.44
	P3x20,h0	183	10	1	109	303	3.6	36.3	*23.61
9 Van Duc A	C,h0	1822	898	101	1293	4114	24.282	33.884	0.00
	P2x0,h0	215	19	170	2447	2851	6.629	91.792	63.90
	P3x0,h0	136	8	142	3003	3289	4.560	95.621	81.60
	P2x20,h0	536	15	221	2266	3038	7.768	81.863	31.50
	P3x20,h0	220	14	164	2273	2671	6.664	91.239	33.60

10 Ladji CQ	C,h0	*89.5	*63.4	*0.6	*23.4	177	36.2	13.6	0.00
	C,h96	*79.1	*150.0	*7.0	*26.9	263	59.7	12.9	0.00
	C,h320	*53.2	*162.8	*7.1	*24.9	248	68.5	12.9	0.00
	α,h0	*110.8	*35.3	*19.7	*21.3	187	29.4	21.9	NA
	α,h96	*99.0	*55.9	*32.2	*14.9	202	43.6	23.3	NA
	α,h320	*69.0	*84.0	*23.9	*11.1	188	57.4	18.6	NA
11 Magugu	C,h1600	40	311	13	44	408	79.4	14.0	0.00
	C,h0	56	61	1	105	223	27.8	47.5	0.00
	NN	44	321.5	25.5	12	403	86.1	9.3	0.00
	p,h0	69	35	40	123	267	28.1	61.0	NA
	p,h1600	75	54	70	121	320	38.8	59.7	NA

Legend:
U = unfed, F = fed, A = alive, D = dead, *p* = insecticide concentration after completion of the experimental hut essays, * = estimated value, C = control net, P2 = PermaNet 2.0, P3 = PermaNet 3.0, x0 = zero washes, x20 = 20 standard WHO washes, h0 = intact (h = 0 cm^2), h96 = holed 6x4x4 cm (h = 96 cm^2), h320 = holed 80x2x2 cm (h = 320 cm^2), h1600= holed 8x20x10 cm (h = 1600 cm^2), p = target concentration 200 mg/m^2 permethrin, α = 40 mg/m^2 target concentration alphacypermethrin, NN = person without a net. Data presented on Magugu excludes gravid mosquitoes.
