# Supplementary material for: Effects of pyrethroid resistance on the cost effectiveness of a mass distribution of long-lasting insecticidal nets: a modelling study
Source: Malar J. 2013 Feb 25;12:77. doi: 10.1186/1475-2875-12-77 (PMC3598792; doi:10.1186/1475-2875-12-77)
Supplement: Additional file 2 — Experiment parameterization and parameter values. [file 1475-2875-12-77-S2.pdf]

Additional file 2. Experiment parameterization and parameter values

In this document, for parameters (italicised) and parameter groups (italicised), where different from the ‘central scenario’ described in the appendix by Briët and colleagues [1], detailed information is given on the choice of the parameter values, organised according to the hierarchical organization of a scenario script.

demography

The ‘Ifakara’ *demography* [2] was used. The population is stationary and approximately stable: individuals move up in age group with time, and because this structure is monotonically decreasing with age, surplus individuals are out-migrated (also above *maximumAgeYrs*).

popSize

A large population size (*popSize*) of 100,000 was used. The estimation of effects of LLINs on DALYs, is largely based on malaria related deaths, which are rare events. For this, a larger population size yields less stochastic results.

monitoring

For this study, the following ouput variables are relevant: *nTreatments1*, *nTreatments2*, *nTreatments3*, *nUncomp*, *nSevere*, *nSeq*, *nHospitalDeaths*, *nIndDeaths*, *nDirDeaths*, *nHospitalRecovs*, *nHospitalSeqs*, and *nMassITNs*. These output variables are used to calculate NHB (See Additional file 3).

interventions > ITN > usage value

The usage value can be interpreted as the probability that host searching occurs during the time that people who own a net are using the net. By setting the usage value to 0.75 (75%), and LLIN effects *deterrency*, *preprandialKillingEffect* and *postprandialKillingEffect* (see elsewhere in this document) equal for both sub-populations (see: *entomology* elsewhere in this document), a mosquito has for each time she finds a host, a 25% chance that this will be during the time that LLIN users would be under the net. This corresponds to the stochastic behaviour setting, which is equivalent to the setting in the central scenario of [1]. By setting the usage value to 1.0, and setting LLIN effects nil for the sub-population named ‘outdoor’, only the population named ‘indoor’ will encounter human hosts only during the time that people who own a net are using the net, whereas the ‘outdoor’ population will never be affected by LLINs.

interventions > ITN > holeRate mean

The level of the annual hole formation rate of nets, the *holeRate mean*, was set at 1.8 holes per net per year. This value was based on re-analysis of the data on distribution of the total number of holes in Olyset nets after seven years of use [3], provided by Christian Lengeler.

interventions > ITN > holeRate sigma

The value of the hole formation rate is varied among nets by multiplying with a distribution factor which is log normally distributed with mean one and the standard deviation of the log transformed variable *sigma*). The distribution factor is generated by taking one sample per net from a Gaussian distribution with mean zero and standard deviation one. For each parameter (*holeRate*, *ripRate*, *insecticideDecay* rate), the same sample is multiplied by the respective sigma and a constant (*mu*) added such that, once exponentiated, the mean of the variable over nets is one. For *insecticideDecay* rate, this constant can be chosen freely. The transformed sample is then exponentiated to obtain the respective distribution factor. This procedure implies that the distribution of *holeRate*, *ripRate* and insecticide decay rate are supposed to be covariant:

nets that are heavily used decay fast both chemically and physically, whereas nets that are gently used decay slowly both chemically and physically. There is some evidence that these are indeed associated [4].
The level of the *sigma* parameter of the distribution factor for hole formation rates was set to 0.8. This value was also based on re-analysis of the raw data on distribution of the total number of holes in Olyset nets after seven years of use [3], provided by Christian Lengeler.

interventions > ITN > ripRate mean

The *ripRate mean* value was set equal to the value of the *holeRate mean*. (The ripping process was assumed to be similar to the hole formation process).

interventions > ITN > ripRate sigma

The *riprate sigma* value was set equal to the value of the *holeRate sigma*. (The ripping process was assumed to be similar to the hole formation process).

interventions > ITN > ripFactor value

The *ripFactor value* expresses how important rips are in increasing the (proportionate) hole index. A net's hole index is the hole count plus the *ripFactor value* multiplied with the cumulative number of rips. With the central values for *holeRate mean*, *ripRate mean*, *holeRate sigma* and *ripRate sigma*, a *ripFactor value* of 0.30 allowed to approximate the upward curve in the mean hole index shown by Kilian and colleagues [5]. Based on this, the level of the *ripFactor value* was set to 0.30.

interventions > ITN > initialInsecticide mu
The mean insecticide content of new nets (*initialInsecticide mu*) was set to 55 mg.m^{-2} (corresponding to 1.8 g active ingredient (AI)/kg for a 75-denier and 1.4 g/kg for a 100-denier net) for P2 or 85.5 mg.m^{-2} (corresponding to 2.8 g AI/kg in the side panels made out of 75-denier netting material) for P3 [6]. The fact that P3 has higher deltamethrin content and PBO in the top panel was ignored. As this was also done for the parameter value calculations of the effects of LLINs (see elsewhere in this document), this should not have important effects on the results.

interventions > ITN > initialInsecticide sigma
The insecticide concentration of new nets is Gaussian distributed. The standard deviation (*sigma*) was set to 14, based on the interquartile range observed by Kilian and colleagues [5], for P2.

interventions > ITN > insecticideDecay L and function
The *insecticideDecay function* chosen was "exponential", $\varphi_t = exp\left(-\frac{t\ln(2)}{L}\right)$ with φ_t the proportion of the initial insecticide concentration remaining at time *t* (in years). The *insecticideDecay L* parameter then directly translates into the insecticide half-life in years. However, if the decay rate $\lambda = \ln(2)/L$ is heterogeneous, the mean half-life is longer. The level of the *insecticideDecay L* for the decay rate of the insecticide in the nets was taken as 1.5, which, if combined with a central distribution factor *insecticideDecay sigma* of 0.8, yields a

mean half-life of about two years. This roughly corresponds to the decay of second generation LLINs [4,5].

interventions > ITN > insecticideDecay sigma (and mu)
The parameters *insecticideDecay mu* and *insecticideDecay sigma* are for the distribution factor (same samples as for the *holeRate* distribution factor). The variation in the insecticide increases over time due to the heterogeneity in the insecticide decay rate. Such behaviour is also apparent from data presented by Killian and colleagues [5]. The level of *insecticideDecay sigma* was chosen at 0.8 and *insecticideDecay mu* was chosen such that the mean was equal to one (*insecticideDecay mu* = −0.32 for the central value).

interventions > ITN > attritionOfNets L and function and k
The attrition function used is "smooth-compact", $\psi_t = exp\left(k - \frac{k}{1-(t/L)^2}\right)$ with ψ_t the proportion of the initial net coverage remaining at time *t* (in years). A *k* value of 18 was used. The smooth-compact function with this *k* value was applied by Nakul Chitnis to data on net ownership provided by Albert Kilian (Chitnis and Kilian, personal communications). The *L* parameter was and chosen such that 50% of nets initially distributed had disappeared after 4 years. This was at an L value of 20.773. It should be noted that from the simulated population, which is kept at a stationary size, people are out-migrated (with their nets) due to population growth. Therefore, the attrition rate of nets per person in the simulated population is slightly higher than the attrition of nets; if the half-life of the attrition of nets would be infinity, with a population growth of 3.47%, the half-life of nets per person in the simulated population would be about 20 years. Population growth may thus explain part of the observed difference in attrition rates between prospective studies (cohort based) and population wide surveys.

interventions > ITN > anophelesParams >preprandialKillingEffect
The estimation of the parameter values for the pre-prandial killing effect was done in six steps.
1) The model for pre-prandial killing of mosquitoes of population j for a net of type k with insecticide concentration p and hole index h (cm^2 holed area) as given by Briët and colleagues [7] can be written:

$$PB\mu_{k,p,h,j} = \begin{array}{l} PB\mu\_baseFactor_j + PB\mu\_holeFactor_j \times \exp\left(-h \times PB\mu\_holeScalingFactor_j\right) + \\ PB\mu\_insecticideFactor_{k,j} \times \left(1-\exp\left(-p \times PB\mu\_insecticideScalingFactor_{k,j}\right)\right) + \\ PB\mu\_interactionFactor_{k,j} \times \exp\left(-h \times PB\mu\_holeScalingFactor_{k,j}\right) \times \\ \left(1-\exp\left(-p \times PB\mu\_insecticideScalingFactor_{k,j}\right)\right) \end{array} \quad \text{(eq1)},$$

with the constraints:

$$0 \le PB\mu\_baseFactor_j + PB\mu\_holeFactor_j \le 1$$

$$0 \le PB\mu\_baseFactor_j + PB\mu\_insecticideFactor_{k,j} \le 1$$

$$0 \le PB\mu\_baseFactor_j + PB\mu\_holeFactor_j + PB\mu\_insecticideFactor_{k,j} + PB\mu\_interactionFactor_{k,j} \le 1.$$

However, in the data available, the holed area h is not varied within huts trials, and $PB\mu\_holeFactor_j$, $PB\mu\_holeScalingFactor_j$,and $PB\mu\_interactionFactor_{k,j}$ are not identifiable. Without these factors, the reduced model looks like:

$$PB\mu_{k,p,h,j} = \begin{matrix} PB\mu\_baseFactor_j + \\ PB\mu\_insecticideFactor_{k,j} \times \left(1 - \exp\left(-p \times PB\mu\_insecticideScalingFactor_{k,j}\right)\right) \end{matrix} \quad \text{(eq2)},$$

with the constraint:
$0 \le PB\mu\_baseFactor_j + PB\mu\_insecticideFactor_{k,j} \le 1.$

Using the three observed data points over the range from untreated, via 20 times washed nets ($p = p[kX\,20]$) to unwashed nets ($p = p[kX\,0]$), the reduced version of the model (eq2) was fitted for $j \in \{1,2,3,4,5,6,7,8,9\}$ and $k \in \{P2, P3\}$ by optimizing the residual sum of squares. In order to make sure that the slope of the curve in the area of low insecticide was not too steep, the $PB\mu\_insecticideScalingFactor$ was also constrained, $PB\mu\_insecticideScalingFactor_{k,j} \le 0.3$.

2) In order to be able to model the decay of the pre-prandial killing effect with physical decay, and to compare the effect found for Yaokoffikro with the other populations, the effect of holed area from the study on *Cx. quinquefasciatus* [8] can be used to estimate the parameter values for the effect of holes in the hut studies.

For population 'Ladji CQ' (Additional file 1 and Table 1), in the untreated net category, the total number of mosquitoes in the intact net (177) was considerably lower than in the arms with 96 cm^2 holes (263) and 320 cm^2 (248) (See Additional File 1). Since untreated nets are not expected to have any deterrent effect on entry into the hut, there must have been a considerable amount of mosquitoes that entered into the hut with intact nets, failed to feed (or die) and escaped without being trapped in the exit traps. This indicates that it is likely that these experimental huts are imperfect in that they still allow mosquitoes to escape.

Assuming that if anything, the escaping would be higher in the 96 cm^2 holed nets than the 320 cm^2 holed nets, the fact that the total number of mosquitoes was slightly higher in the 96 cm^2 holed nets was attributed to stochastic noise, and it was assumed that the difference in escaping of unfed alive mosquitoes was negligible between the two net types.

In order to calculate the proportion of attacking mosquitoes in the untreated intact net arm, the unfed alive category was inflated so that the total number of mosquitoes was the average of the total caught with 96 cm^2 holed type nets and 320 cm^2 holed type nets (263+248)/2=255.5.

The proportion of mosquitoes attacking out of those estimated to have entered was then 34.3% (34.1–34.4) for the intact untreated net, 69.9 (69.7–70.2)% for the 96 cm2 holed area net, and 78.6% (78.5–78.7) for the 320 cm^2 holed net.

For the treated net category, the total of mosquitoes caught in the arm with intact nets was very similar to the total in the arm with the 320 cm^2 holed area, so there, the number of unfed alive mosquitoes was not inflated for the intact treated net arm.

Subsequently, the number of fed dead mosquitoes in each arm was estimated using two different methods, and the results were averaged. In the first method, the number of fed dead mosquitoes is calculated as the number of feds multiplied by the proportion of fed dead mosquitoes out of the number of fed mosquitoes in the corresponding net type in Magagugu, and the second method was the number of dead mosquitoes multiplied by the proportion of fed dead mosquitoes out of the number of dead mosquitoes in the corresponding net type in Magagugu.

Treated holed nets (1600 cm^2) in Magugu (Additional file 1 and Table 1) were assumed to correspond to treated and holed nets in Ladji (both 96 and 320 cm^2 holed area). With the aid of the estimate for the number of dead mosquitoes, the numbers of mosquitoes in the other three insect state categories were estimated.

The proportions attacking for treated nets were 40.8% (35.6–45.9), 51% (43.6–58.4), and 63.3% (57.4–69.2) for 0, 96 and 320 cm^2 holed area nets. The data in brackets give the range. This was estimated, since the data were not available in the four categories.

Table 1: Additional data sources

Population		Start	Lat.	Lon.	AG (%)	deltamethrin 0.05%		permethrin 0.75%		Ref.
						† (%)	n	† (%)	n	
10	Ladji CQ	Aug-07	6.38	2.42	CQ					[8]
11	Magugu	1984	-4.02	35.77	<50[a]			>91.0[b]		[17,18]

Legend

Lat.: latitude; Lon.: longitude; AG: *Anopheles gambiae* sensu stricto, the complement is *Anopheles arabiensis*, unless CQ is indicated; Ref.: Reference; †: mortality; n: (approximate) number of mosquitoes tested for one hour in WHO susceptibility (tube) tests with insecticide on filter papers [20]; CQ: *Culex quinquefasciatus*; [a]Based on the statement "mainly arabiensis" in Lines and colleagues [15]; [b]Lines and colleagues [47] reported on average 91% mortality in one minute WHO cone tests on freshly impregnated and one month old mosquito netting with a target permethrin dose of 200 mg.m^{-2}. Based on mortality estimations of >80% in WHO susceptibility tests with 0.25% permethrin on filter paper after one hour exposure *versus* <20% mortality in WHO cone tests with 500 mg.m^{-2} permethrin on netting after 1 minute exposure for mosquito populations in Kenya [48], it can be safely assumed that mortality of population 'Magugu' would be greater than 91% in one hour susceptibility tests with 0.75% permethrin on filter paper.

For each of the six arms for Ladji CQ, the pre-prandial killing probability was calculated, and the parameters of the following function were estimated by minimizing the residual sum of squares of the following function, which is the full function excluding an insecticide scaling factor:

$$PB\mu_{k,p,h,j} = \begin{array}{l} PB\mu\_baseFactor_j + holeFactor_j \times \exp\left(-h \times PB\mu\_holeScalingFactor_j\right) + \\ PB\mu\_insecticideFactor_{k,j} \times p + \\ PB\mu\_interactionFactor_{k,j} \times \exp\left(-h \times PB\mu\_holeScalingFactor_j\right) \times p \end{array} \quad \text{(eq. 3)},$$

with the constraints:

$$0 \le PB\mu\_baseFactor_j + PB\mu\_holeFactor_j \le 1$$

$$0 \le PB\mu\_baseFactor_j + PB\mu\_insecticideFactor_{k,j} \le 1$$

$$0 \le PB\mu\_baseFactor_j + PB\mu\_holeFactor_j + PB\mu\_insecticideFactor_{k,j} + PB\mu\_interactionFactor_{k,j} \le 1,$$

and with $p = 0$ for untreated nets and $p = 1$ for treated nets.

With the parameters estimated, the preprandial killing effects for nets with no insecticide ($p = 0$) and $h \in \{0, 96, \inf \approx 10\exp 5\}$, thus $\hat{PB}\mu_{\mathrm{k=C},p=0,h=0,j=10}$, $\hat{PB}\mu_{\mathrm{k=C},p=0,h=96,j=10}$ and, $\hat{PB}\mu_{\mathrm{k=C},p=0,h=\mathrm{inf},j=10}$, were estimated.

Similarly, assuming that at $p = 1$ the insecticide effect was at its asymptote, also, $\hat{PB}\mu_{\mathrm{k=C},p=\mathrm{inf},h=0,j=10}$ and $\hat{PB}\mu_{\mathrm{k=C},p=\mathrm{inf},h=96,j=10}$ were estimated. These were transformed to logit values, using the formula:

$$\mathrm{logit}(\alpha) = \log\left(\frac{\alpha}{1-\alpha}\right).$$

Next, for all populations except Yaokoffikro, thus for $j \in \{1,2,3,4,5,6,7,9\}$, the preprandial killing for intact nets without insecticide was estimated as the inverse-logit value ($\mathrm{inv.logit}(\beta) = \dfrac{\exp(\beta)}{1+\exp(\beta)}$) of the sum of the logit of the pre-prandial killing at $h = 96$ and the

difference between the logit values for untreated nets:

$$\hat{PB\mu}_{\text{k=C},p=0,h=0,j=j} = \text{inv.logit}\left(\text{logit}\left(PB\mu_{k=C,p=0,h=96,j=j}\right) + \text{logit}\left(\hat{PB\mu}_{\text{k=C},p=0,h=0,j=10}\right) - \text{logit}\left(\hat{PB\mu}_{\text{k=C},p=0,h=96,j=10}\right)\right)$$

$$\hat{PB\mu}_{\text{k=C},p=0,h=\text{inf},j=j} = \text{inv.logit}\left(\text{logit}\left(PB\mu_{k=C,p=0,h=96,j=j}\right) + \text{logit}\left(\hat{PB\mu}_{\text{k=C},p=0,h=\text{inf},j=10}\right) - \text{logit}\left(\hat{PB\mu}_{\text{k=C},p=0,h=96,j=10}\right)\right)$$

For Yaokoffikro ($j = 8$), the pre-prandial killing effect at 96 cm^2 holed area ($h = 96$) and at an infinite holed area ($h = \text{inf}$) was calculated as:

$$\hat{PB\mu}_{\text{k=C},p=0,h=\text{inf},j=j} = \text{inv.logit}\left(\text{logit}\left(PB\mu_{k=C,p=0,h=0,j=j}\right) + \text{logit}\left(\hat{PB\mu}_{\text{k=C},p=0,h=\text{inf},j=10}\right) - \text{logit}\left(\hat{PB\mu}_{\text{k=C},p=0,h=0,j=10}\right)\right).$$

3) Using the two estimated values in step 2 and one observed value for pre-prandial killing effect over three holed area values $h \in \{0, 96, \text{inf} \approx 10\exp 5\}$ in untreated nets ($p = 0$), thus for $j \in \{1,2,3,4,5,6,7,11\}$: $\hat{PB\mu}_{\text{k=C},p=0,h=0,j=j}$, $PB\mu_{\text{k=C},p=0,h=96,j=j}$ and, $\hat{PB\mu}_{\text{k=C},p=0,h=\text{inf},j=j}$, and for $j = 8$: $PB\mu_{\text{k=C},p=0,h=0,j=8}$, $\hat{PB\mu}_{\text{k=C},p=0,h=96,j=8}$ and, $\hat{PB\mu}_{\text{k=C},p=0,h=\text{inf},j=8}$, the $PB\mu\_baseFactor_j$, $PB\mu\_holeFactor_j$, and $PB\mu\_holeScalingFactor_j$ were estimated by fitting a reduced variant of model to the data by optimizing the residual sum of squares:

$$PB\mu_{k=C,p=0,h,j} = PB\mu\_baseFactor_j + PB\mu\_holeFactor_j \times \exp\left(-h \times PB\mu\_holeScalingFactor_j\right)$$

(eq. 4), with the constraint:

$0 \le PB\mu\_baseFactor_j + PB\mu\_holeFactor_j \le 1$.

In order to make sure that the slope of the curve in the area of low holed area was not too steep, the $PB\mu\_holeScalingFactor$ was also constrained, i.e.: $PB\mu\_holeScalingFactor_j \le 0.2$.

This model does not estimate the $PB\mu\_insecticideFactor_{k,j}$, $PB\mu\_insecticideScalingFactor_{k,j}$ and the $PB\mu\_interactionFactor_{k,j}$ because these do not play a role for untreated nets.

4) The model and fitted parameter values in step 1 (eq2) were then used to estimate the pre-prandial killing effect at a high insecticide concentration that would approximate the asymptote, $p = 10^5 \approx \text{inf}$, for $j \in \{1,2,3,4,5,6,7,9\}$ $\hat{PB\mu}_{\text{k=k},p=\text{inf},h=96,j=j}$ and for $j = 8$, $\hat{PB\mu}_{\text{k=k},p=\text{inf},h=0,j=j}$ were estimated, for $k \in \{P2, P3\}$.

Then, the pre-prandial killing effect at infinitely high insecticide concentration and the holed area tested was estimated. For $j \in \{1,2,3,4,5,6,7,9\}$:

$$\hat{PB\mu}_{\text{k=k},p=\text{inf},h=0,j=j} = \text{inv.logit}\left(\text{logit}\left(\hat{PB\mu}_{\text{k=k},p=\text{inf},h=96,j=j}\right) + \text{logit}\left(\hat{PB\mu}_{\text{k=k},p=\text{inf},h=0,j=10}\right) - \text{logit}\left(\hat{PB\mu}_{\text{k=k},p=\text{inf},h=96,j=10}\right)\right),$$

and for $j = 8$:

$$\hat{PB\mu}_{\text{k=k},p=\text{inf},h=96,j=j} = \text{inv.logit}\left(\text{logit}\left(\hat{PB\mu}_{\text{k=k},p=\text{inf},h=0,j=j}\right) + \text{logit}\left(\hat{PB\mu}_{\text{k=k},p=\text{inf},h=96,j=10}\right) - \text{logit}\left(\hat{PB\mu}_{\text{k=k},p=\text{inf},h=0,j=10}\right)\right).$$

5) The reduced version of the model as given in the equation (eq3) under step 2, without $PB\mu\_insecticideScalingFactor_{k,j}$, was used to estimate the $PB\mu\_interactionFactor_{k,j}$, assuming fixed values for the $PB\mu\_baseFactor_j$, $PB\mu\_holeFactor_j$ and $PB\mu\_holeScalingFactor_j$ as fitted using the equation (eq4) in step 3. This model (eq3) was fit to the two estimated data points in

step 4: $\hat{PB\mu}_{\mathrm{k=k},p=\mathrm{inf},h=0,j=j}$ and $\hat{PB\mu}_{\mathrm{k=k},p=\mathrm{inf},h=96,j=j}$.

6) The full version of the model, as given in the first equation in step 1 (eq1), was used to estimate the parameter values for the $PB\mu\_insecticideScalingFactor_{k,j}$, assuming fixed values for the $PB\mu\_baseFactor_j$, $PB\mu\_holeFactor_j$ and $PB\mu\_holeScalingFactor_j$ as fitted using the equation (eq4) in step 3, and the $PB\mu\_interactionFactor_{k,j}$, as estimated in step 5.

This model was fitted to the three observed data points for each population and net type:

for $j \in \{1,2,3,4,5,6,7,9\}$: $PB\mu_{\mathrm{k=C},p=0,h=96,j=j}$, $PB\mu_{\mathrm{k=k},p=p[kX20],h=96,j=j}$ and, $PB\mu_{\mathrm{k=k},p=p[kX0],h=96,j=j}$, and

for $j = 8$: $PB\mu_{\mathrm{k=C},p=0,h=0,j=j}$, $PB\mu_{\mathrm{k=k},p=p[kX20],h=0,j=j}$ and, $PB\mu_{\mathrm{k=k},p=p[kX0],h=0,j=j}$.

Parameter estimates are given in Table 2.

Figure 1 allows a comparison of the observed pre-prandial killing effect with the fitted relationship between the pre-prandial killing effect and insecticide concentration. Note that the fitted curve goes in general through the three points, except where the third point is lower than the second (e.g. P3 for population Yaokoffikro).

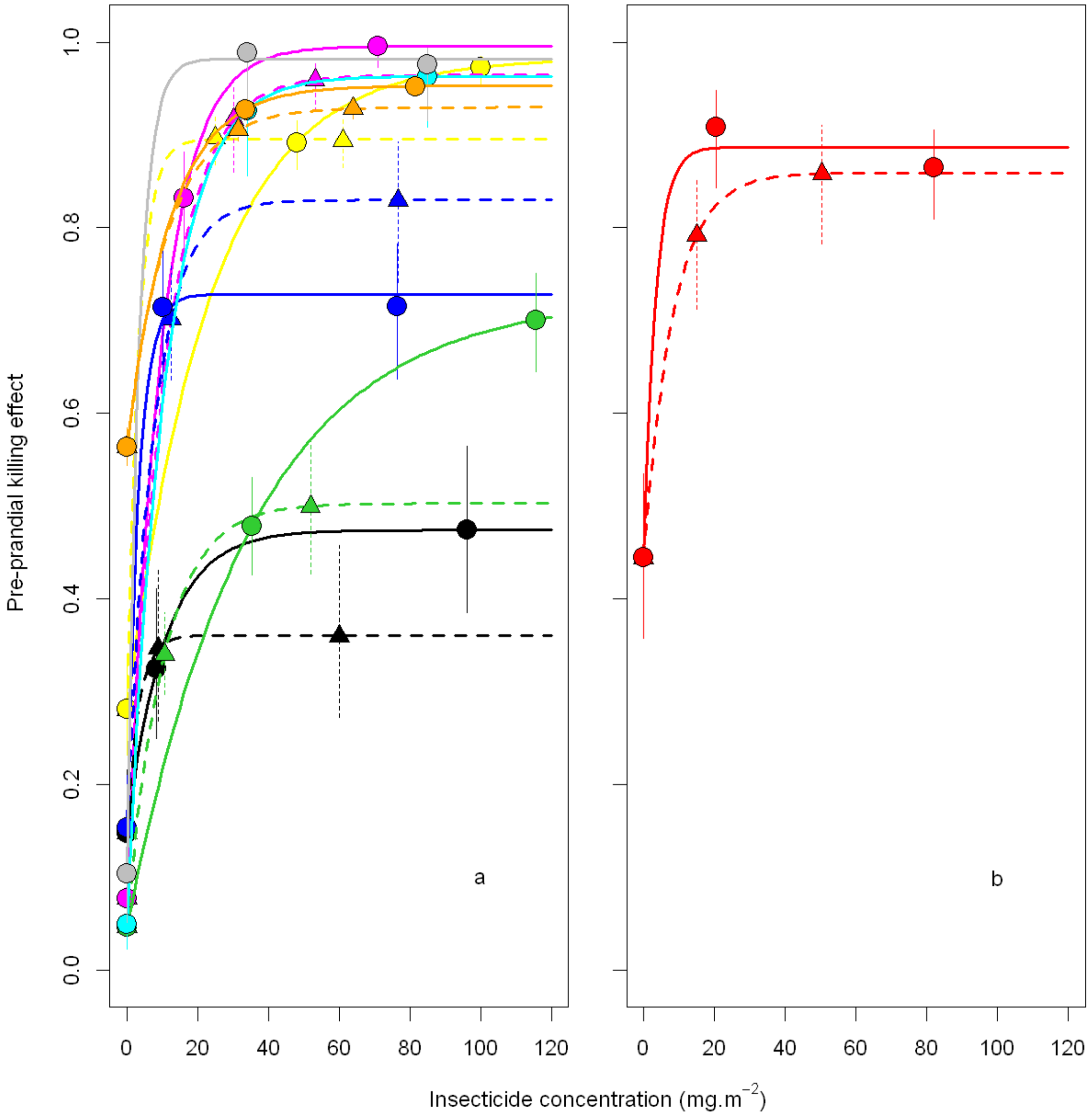

Figure 1. Pre-prandial killing effect depending on insecticide concentration. Relationship of the pre-prandial killing effect with insecticide concentration **a**) for nets with a holed area of 96 cm^2; **b**) for intact nets. Circles and triangles represent observations for PermaNet 3.0 and PermaNet 2.0, respectively. Thin solid and interrupted vertical lines represent 95% confidence intervals for PermaNet 3.0 and PermaNet 2.0, respectively. Thick solid and interrupted curved lines represent fitted relationships for PermaNet 3.0 and PermaNet 2.0, respectively. Colours represent mosquito populations 1–9 in Table 1, with black: 'Akron', red: 'Yaokoffikro', lime green: 'Kou', orange: 'Van Duc A', dark blue: 'Pitoa', cyan: 'New Bussa AG', magenta: 'Malanville', yellow: 'Zeneti', and grey: 'New Bussa AA'. Note that circles and triangles for the same population overlap at 0 insecticide, as these observations are for untreated nets.

interventions > ITN > anophelesParams >postprandialKillingEffect

In the experimental hut data [8] on *Cx. quinquefasciatus* in Ladji (population 10), no clear relationship was found between the holed area and the post-prandial killing effect. Therefore, it was assumed that holed area did not influence post-prandial killing effect, and the estimation of the parameter values for the post-prandial killing effect was done in only one step, which is similar to step 1 for the pre-prandial killing effect. Parameter estimates are given in Table 2.

Figure 2 allows a comparison of the observed pre-prandial killing effect with the fitted relationship between the post-prandial killing effect and insecticide concentration. Note that the fitted curve goes in general through the three points, except where the third point is lower than the second (e.g., P2 for population Malanville). Also, the curve for P3 in Malanville does not go through the third point. This is because the post-prandial killing effect is restricted to be equal or smaller than one.

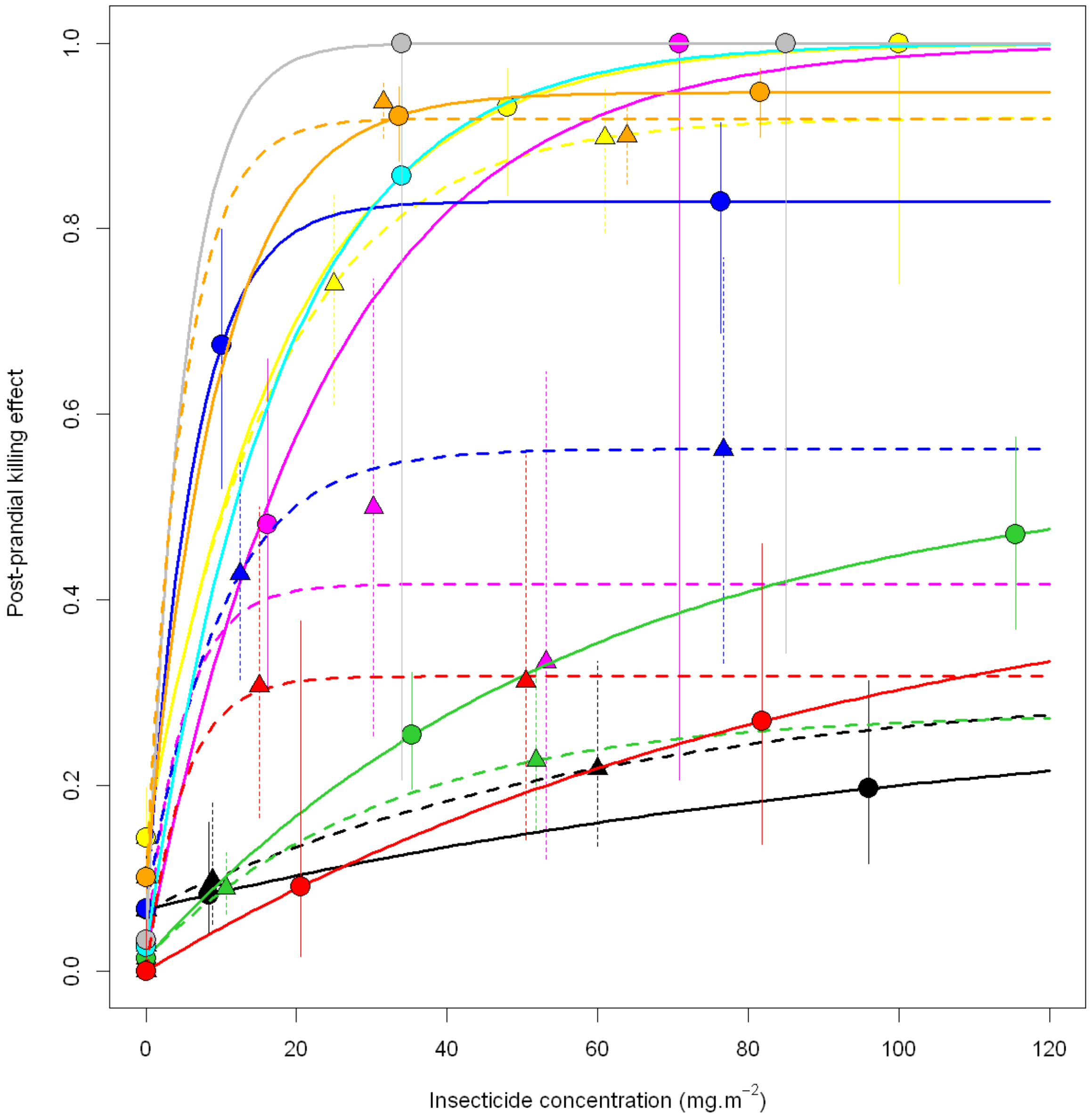

Figure 2. Post-prandial killing effect depending on insecticide concentration. Relationship of the post-prandial killing effect with insecticide concentration. Legend as in Figure 1.

interventions > ITN > anophelesParams > twoStageDeterrency > entering

Briët and colleagues [7] used the term "one minus the relative number of affected mosquitoes ($RA_{1\text{ vs }2}$)" to define deterrency of a host of type 1, as compared to another host of type 2. The relative number of directly affected mosquitoes is the same as the ratio of the number of mosquitoes attacking, because all mosquitoes that attack are supposed to either die or blood feed, or both, in the process. In this work, deterrency is redefined as one minus the relative proportion of hut entry, where the probability of hut entry, $Pent_{k,p,j}$, of mosquitoes of population j for a net of type k with insecticide concentration p was assumed not to depend on the holed area of the net:

$$Pent_{k,p,j} = \; exp\left(\log\left(Pent\_insecticideFactor_{k,j}\right)\times\left(1-\exp\left(-p\times Pent\_insecticideScalingFactor_{k,j}\right)\right)\right)$$

(eq5).

with the constraint $0 \le Pent\_insecticideFactor_{k,j} \le 1$.

Using the three observed data points over the range from untreated, via 20 times washed nets ($p = p\left[kX\,20\right]$) to unwashed nets ($p = p\left[kX\,0\right]$), this model (eq5) was fitted for $j \in \{1,2,3,4,5,6,7,8,9\}$ and $k \in \{P2, P3\}$ by optimizing the residual sum of squares.

In order to make sure that the slope of the curve in the area of low insecticide was not too steep, the $Pent\_insecticideScalingFactor$ was also constrained, $Pent\_insecticideScalingFactor_{k,j} \le 0.2$. Parameter estimates are given in Table 2.

Figure 3 allows a comparison of the observed probability of hut entry with the fitted relationship between the probability of hut entry and insecticide concentration. Note that the fitted curve goes in general through the three points, except where the third point is higher than the second (e.g., Yaokoffikro for P2 and P3, Malanville for P2, and New Bussa AG for P3).

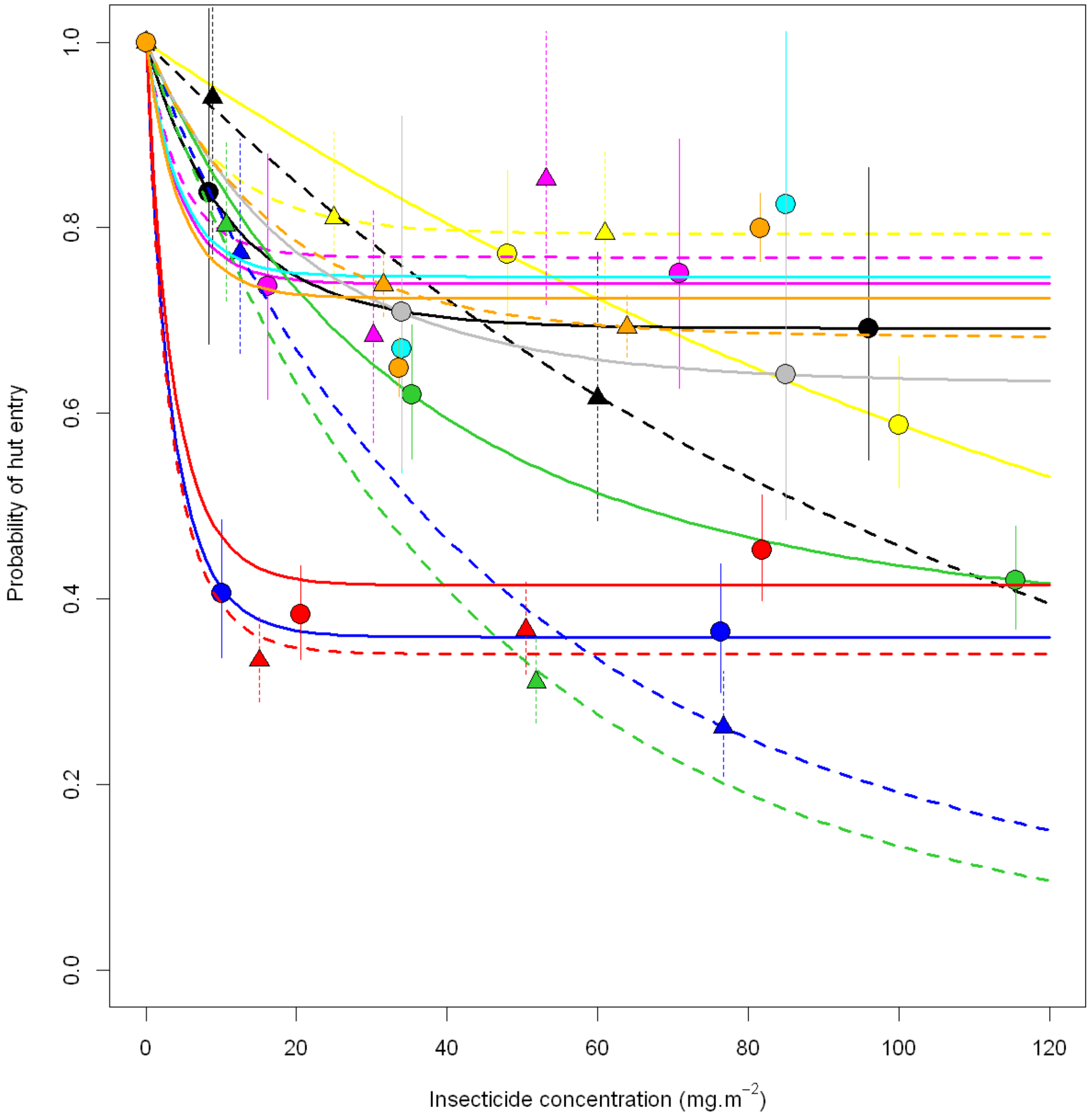

Figure 3. Probability of hut entry depending on insecticide concentration. Relationship of the probability of hut entry with insecticide concentration. Legend as in Figure 1.

interventions > ITN > anophelesParams > twoStageDeterrency > attacking

The probability of attacking conditional on having entered the hut (in a hut trial, the proportion out of all mosquitoes found in the hut that are fed or dead), $Patt_{k,p,h,j}$, for mosquitoes of population j for a net of type k with insecticide concentration p and holed area h was assumed to follow a similar equation to that described for the pre-prandial killing effect (eq1):

$$Patt_{k,p,h,j} = \begin{array}{l} Patt\_baseFactor_j + Patt\_holeFactor_j \times \exp\left(-h \times Patt\_holeScalingFactor_j\right) + \\ Patt\_insecticideFactor_{k,j} \times \left(1 - \exp\left(-p \times Patt\_insecticideScalingFactor_{k,j}\right)\right) + \\ Patt\_interactionFactor_{k,j} \times \exp\left(-h \times Patt\_holeScalingFactor_{k,j}\right) \times \\ \left(1 - \exp\left(-p \times Patt\_insecticideScalingFactor_{k,j}\right)\right) \end{array} \quad \text{(eq6)},$$

with the constraints:

$$0 \le Patt\_baseFactor_j + Patt\_holeFactor_j \le 1$$

$$0 \le Patt\_baseFactor_j + Patt\_insecticideFactor_{k,j} \le 1$$

$$0 \le Patt\_baseFactor_j + Patt\_holeFactor_j + Patt\_insecticideFactor_{k,j} + Patt\_interactionFactor_{k,j} \le 1$$

The ratio of the number of mosquitoes attacking with a host of type 1 compared to a host of type 2 can be calculated by multiplying the probability of hut entry and the probability of attacking conditional on having entered: $\mathrm{RA}_{\mathrm{k=k[1],p=p[1]},h=h[1]\ \mathrm{vs\ k=k[2],p=p[2]},h=h[2],j} = \frac{Pent_{k=1,p=p[1],j}}{Pent_{k=2,p=p[2],j}} \times \frac{Patt_{k=1,p=p[1],h=h[1],j}}{Patt_{k=2,p=p[2],h=h[2],j}}$.

The estimation of the parameter values is, like with $PB\mu_{k,p,h,j}$, described in six steps. Like with $PB\mu_{k,p,h,j}$, in the data available, the holed area h is not varied within huts trials, the $Patt\_holeFactor_j$, $Patt\_holeScalingFactor_j$ and $Patt\_interactionFactor_{k,j}$ are not identifiable. Without these factors, the reduced model for the probability of attacking looks like:

$$Patt_{k,p,h,j} = \begin{array}{l} Patt\_baseFactor_j + \\ Patt\_insecticideFactor_{k,j} \times \left(1 - \exp\left(-p \times Patt\_insecticideScalingFactor_{k,j}\right)\right) \end{array} \quad \text{(eq7)},$$

with the constraint $0 \le Patt\_baseFactor_j + Patt\_insecticideFactor_{k,j} \le 1$.

If the $Patt\_insecticideScalingFactor_{k,j}$ is large, this results in a steep slope of curve at small values of p. This, in turn, may lead the curve for personal protection $PP_{k,p,h,j}$ against p to be non-monotonically increasing or even negative near small values of p, which is unrealistic. Personal protection can be calculated as

$$PP_{\mathrm{k=k,p=p},h=h\ \mathrm{vs\ k=C,p=0},h=\mathrm{inf},j} = \frac{bites_{k=C,p=0,h=\mathrm{inf},j=j} - bites_{k=k,p=p,h=h,j=j}}{bites_{k=C,p=0,h=\mathrm{inf},j=j}} = 1 - \frac{bites_{k=k,p=p,h=h,j=j}}{bites_{k=C,p=0,h=\mathrm{inf},j=j}}$$

$$= 1 - \frac{Pent_{k=k,p=p,j=j}}{Pent_{k=C,p=0,j=j}} \times \frac{Patt_{k=k,p=p,h=h,j=j}}{Patt_{k=C,p=0,h=\mathrm{inf},j=j}} \times \frac{1 - PB\mu_{k=k,p=p,h=h,j=j}}{1 - PB\mu_{k=C,p=0,h=\mathrm{inf},j=j}}$$

$$= 1 - \mathrm{RA}_{\mathrm{k=k,p=p},h=h\ \mathrm{vs\ k=C,p=0},h=\mathrm{inf},j} \times \frac{1 - PB\mu_{k=k,p=p,h=h,j=j}}{1 - PB\mu_{k=C,p=0,h=\mathrm{inf},j=j}}$$

Assuming that nets cannot prevent more bites than received without a net, and do not lead to more bites, $0 \le PP_{\mathrm{k=k,p=p},h=h\ \mathrm{vs\ k=C,p=0},h=\mathrm{inf},j} \le 1$. This constraint is valid if

$$0 \le \mathrm{RA}_{\mathrm{k=k,p=p},h=h\ \mathrm{vs\ k=C,p=0},h=\mathrm{inf},j} \le \frac{1 - PB\mu_{k=C,p=0,h=\mathrm{inf},j=j}}{1 - PB\mu_{k=k,p=p,h=h,j=j}}.$$

The function for personal protection is monotonically increasing if

$$\frac{\partial PB\mu_{k,p,h,j}/\partial p}{1-PB\mu_{k,p,h,j}} \geq \frac{\partial \mathrm{RA}_{\mathrm{k=k,p=p,}h=h\ \mathrm{vs\ k=C,p=0,}h=\mathrm{inf,}j}/\partial p}{\mathrm{RA}_{\mathrm{k=k,p=p,}h=h\ \mathrm{vs\ k=C,p=0,}h=\mathrm{inf,}j}}$$, with

$$\frac{\partial PB\mu_{k,p,h,j}/\partial p}{1-PB\mu_{k,p,h,j}} = \begin{array}{l} PB\mu\_insecticideFactor_{k,j} \times PB\mu\_insecticideScalingFactor_{k,j} \times \\ \exp\left(-p \times PB\mu\_insecticideScalingFactor_{k,j}\right) \times \frac{1}{1-PB\mu_{k=k,p=p,h=h,j=j}} \end{array}$$ and

$$\frac{\partial \mathrm{RA}_{\mathrm{k=k,p=p,}h=h\ \mathrm{vs\ k=C,p=0,}h=\mathrm{inf,}j}/\partial p}{\mathrm{RA}_{\mathrm{k=k,p=p,}h=h\ \mathrm{vs\ k=C,p=0,}h=\mathrm{inf,}j}} = \begin{array}{l} Patt\_insecticideFactor_{k,j} \times Patt\_insecticideScalingFactor_{k,j} \times \\ \exp\left(-p \times Patt\_insecticideScalingFactor_{k,j}\right) \times \frac{1}{Patt_{k=k,p=p,h=h,j=j}} + \\ Pent\_insecticideScalingFactor_{k,j} \times \log\left(Pent\_insecticideFactor_{k,j}\right) \times \\ \exp\left(-p \times Pent\_insecticideScalingFactor_{k,j}\right) \end{array}.$$

Further, in order to make sure that the slope of the curve in the area of low insecticide was not too steep (if not already constrained by the requirements for the personal protection), the $Patt\_insecticideScalingFactor$ was also constrained, $Patt\_insecticideScalingFactor_{k,j} \leq 0.2$.
Using the three observed data points over the range from untreated, via 20 times washed nets ($p = p[kX20]$) to unwashed nets ($p = p[kX0]$), the model (eq7) was fitted for $j \in \{1,2,3,4,5,6,7,8,9\}$ and $k \in \{P2, P3\}$ by optimizing the residual sum of squares, with all constraints as described above.
Steps 2 – 6 of the parameter value estimation were as described for the pre-prandial killing effect.

Figure 4 allows a comparison of the observed probability of attacking with the fitted relationship between the probability of the probability of attacking and insecticide concentration. Note that only for three population and net combinations, the fitted curve goes through the three points (Pitoa for P2, and Malanville and New Bussa AA for P3). Where the level of the third point is in between that of the first and the second (Yaokoffikro and Kou for P2, and Zeneti and New Bussa AG for P3), and where the level of the first point (for an untreated net) is in between the level for unwashed and 20 times washed nets (Kou with P3 nets and population Akron for P2 nets), the fit is less close because such a non-monotonic relationship is not allowed. For one combination (Malanville P2), the curve is restricted not to be larger than one. Finally, for four population and net combinations (Yaokoffikro, Akron and Pitoa for P3, and Zeneti for P2), the fitted curves are not fitting well because the parameter estimation model is restricted to the curve for the personal protection against insecticide concentration increasing monotonically. Parameter estimates are given in Table 2.

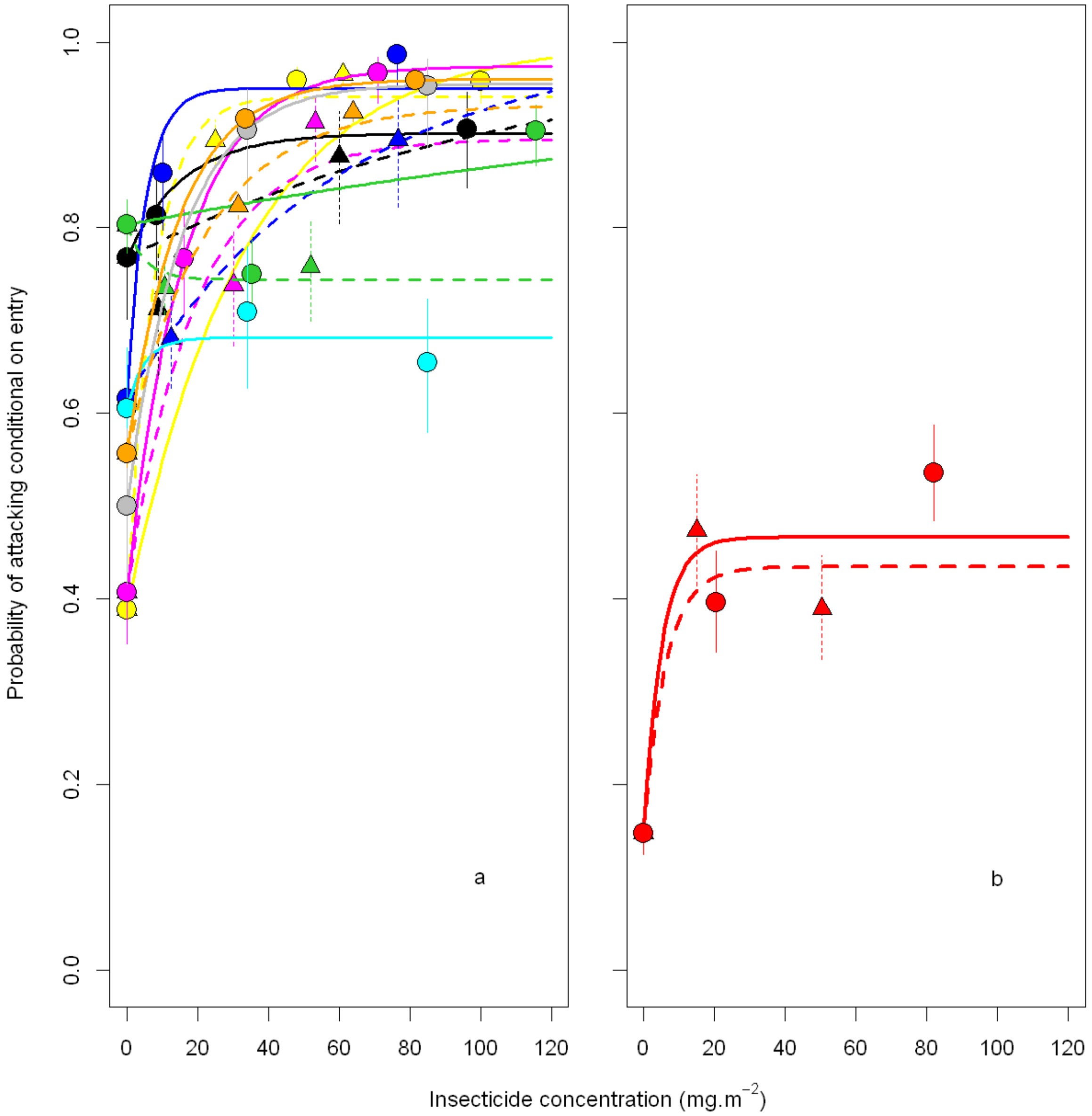

Figure 4. Probability of attacking depending on insecticide concentration. Relationship of the probability of attacking with insecticide concentration **a**) for nets with a holed area of 96 cm^2; **b**) for intact nets. Legend as in Figure 1.

Figure 5 shows the comparison of personal protection relative to untreated control (calculated based on fits to entry, the probability of attacking given entry, and the pre-prandial killing probability) and observed data. Note that the observed personal protection is in general not far off the calculated curve, except for Yaokoffikro with P3 nets, where the observed personal protection was lower with an unwashed net than with a 20 times washed net.

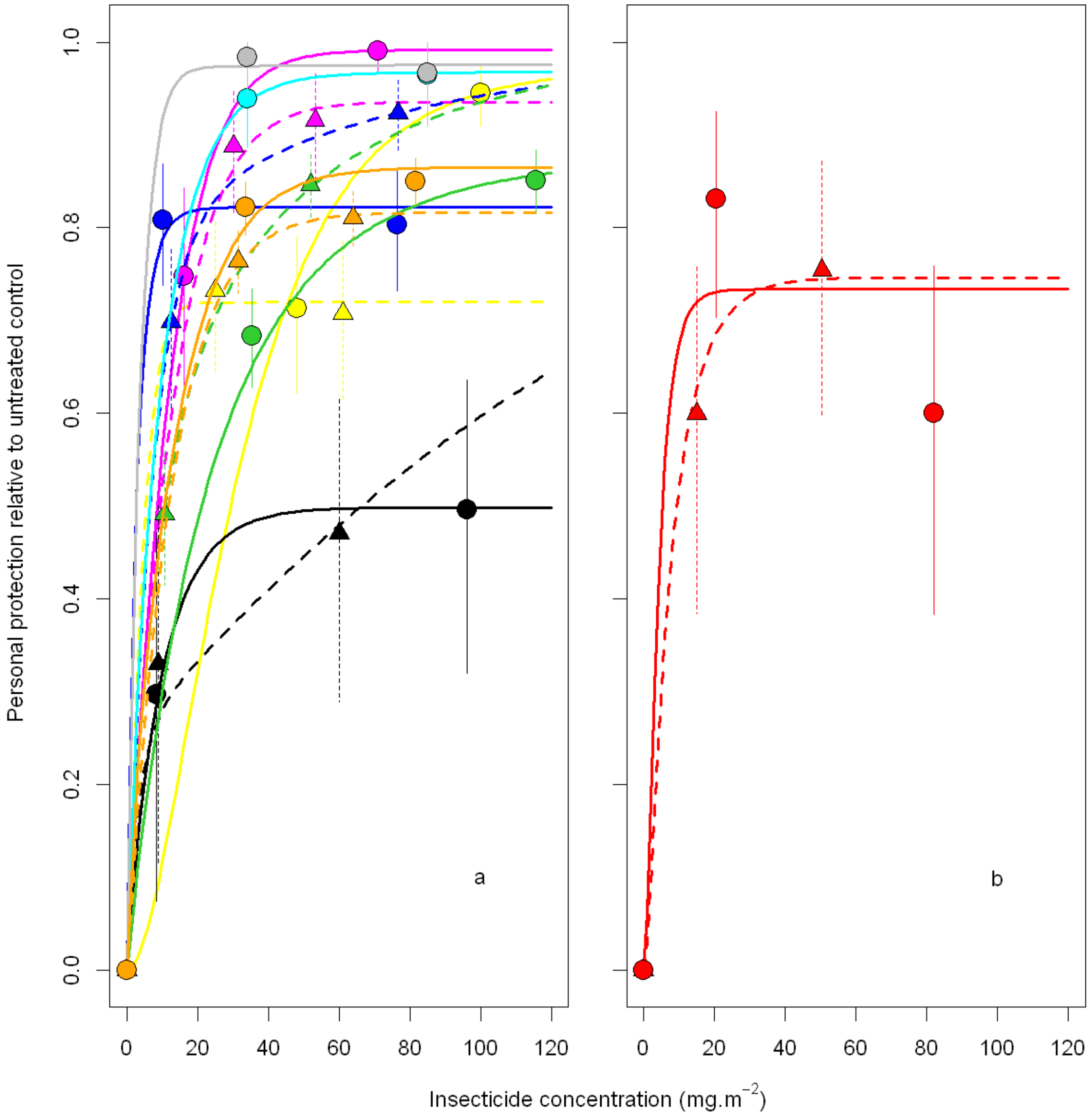

Figure 5. Personal protection relative to an untreated control, depending on insecticide concentration. Relationship of personal protection relative to an untreated control, with insecticide concentration **a**) for nets with a holed area of 96 cm^2; **b**) for intact nets. Legend as in Figure 1.

The corrected mortality (calculated based on fits to entry, the probability of attacking given entry, the pre-prandial killing probability and the post-prandial killing probability), relative to an untreated control, as a function of insecticide concentration, is plotted in Figure 6. In general, the observed corrected mortality is not far off the calculated curve, except for Yaokoffikro and Zeneti with P3 nets, and Malanville with P2 nets. Note that the curve is not monotonic increasing in many population and net combinations. The corrected mortality can decrease with

increasing insecticide concentration as mosquitoes are deterred from entering, and are thus not killed directly by the insecticide. This effect was particularly strong for Kou and Pitoa with P2 nets.

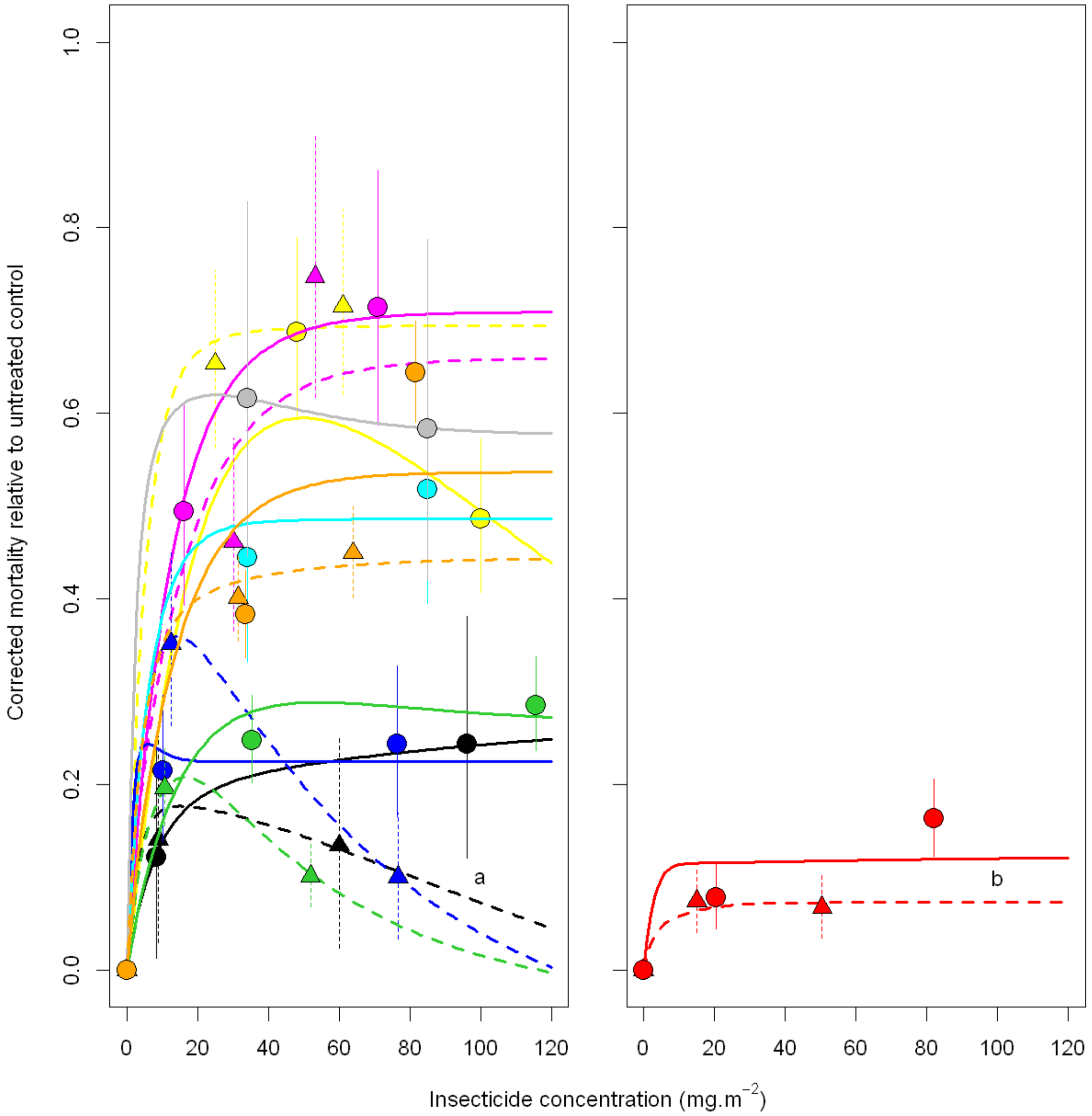

Figure 6. Corrected mortality relative to an untreated control, depending on insecticide concentration. Relationship of corrected mortality relative to an untreated control, with insecticide concentration **a**) for nets with a holed area of 96 cm^2; **b**) for intact nets. Legend as in Figure 1.

interventions > ITN > timed coverage

The central level of the coverage, which describes the proportion of people that receive a net during mass distribution of nets, was 0.7 (70%). For a sub-experiment, it was set to 0.5 and 0.9.

interventions > importedInfections

From time step zero onwards, 10 infections per 1,000 population per year were imported by stochastically infecting individuals in the population. This was done to ensure that malaria

would not be eliminated from the simulated population, which might overestimate the protective effect of an intervention.

Thus, even if an intervention provides full protection to the entire population, 1% of the population will be infected once per year. These infections do not necessarily develop into disease episodes. These could be seen as infections obtained while travelling to a malarious area.

healthSystem
The “Tanzania ACT” health system was used, described elsewhere [9].

entomology > annualEIR
The pre-intervention EIR was varied over 1, 2, 4, 8, 16, 32, 64, 128, and 264 IBPAPA.

entomology > mode and name
Instead of three different species as modelled in [1], only one species was modelled with the parameterization of *An. gambiae* s.s. in [1]. However, this species was separated into two sub-populations, one named ‘indoor’ and one named ‘outdoor’. The proportion of the ‘indoor’ population was varied between 60, 75 and 90%.

Table 2 Estimated parameter values

	1		2		3		5		4		6	7	8		9	
Population	Pitoa		Kou		Akron		Zeneti		Malan-ville		New Bussa AG	New Bussa AA	Yao-koffi-kro		Van Duc A	
Net type	P2	P3	P2	P3	P2	P3	P2	P3	P2	P3	P3	P3	P2	P3	P2	P3
entering_insecticideFactor	0.018	0.359	0.001	0.378	0.001	0.692	0.768	0.740	0.793	0.001	0.747	0.633	0.341	0.415	0.682	0.724
entering_insecticideScalingFactor	0.005	0.200	0.003	0.019	0.001	0.078	0.200	0.200	0.095	0.001	0.200	0.041	0.200	0.200	0.050	0.200
attacking_baseFactor	0.735	0.735	0.876	0.876	0.851	0.851	0.543	0.543	0.524	0.524	0.726	0.634	0.581	0.581	0.685	0.685
attacking_holeFactor	-0.477	-0.477	-0.406	-0.406	-0.434	-0.434	-0.413	-0.413	-0.403	-0.403	-0.477	-0.455	-0.433	-0.433	-0.471	-0.471
attacking_holeScalingFactor	0.014	0.014	0.018	0.018	0.017	0.017	0.012	0.012	0.011	0.011	0.014	0.013	0.012	0.012	0.014	0.014
attacking_insecticideFactor	0.264	0.226	-0.107	0.123	0.148	0.065	0.383	0.440	0.436	0.476	-0.002	0.334	0.048	0.079	0.263	0.285
attacking_insecticideScalingFactor	0.017	0.189	0.200	0.004	0.009	0.063	0.052	0.062	0.133	0.030	0.200	0.065	0.161	0.198	0.045	0.067
attacking_interactionFactor	0.476	0.434	0.268	0.406	0.433	0.361	0.322	0.387	0.346	0.402	0.309	0.414	0.239	0.240	0.412	0.435
preprandialKillingEffect_baseFactor	0.121	0.121	0.036	0.036	0.116	0.116	0.060	0.060	0.228	0.228	0.038	0.081	0.233	0.233	0.494	0.494
preprandialKillingEffect_holeFactor	0.145	0.145	0.053	0.053	0.141	0.141	0.084	0.084	0.210	0.210	0.056	0.108	0.212	0.212	0.226	0.226
preprandialKillingEffect_holeScalingFactor	0.015	0.015	0.016	0.016	0.015	0.015	0.016	0.016	0.014	0.014	0.016	0.016	0.014	0.014	0.012	0.012
preprandialKillingEffect_insecticideFactor	0.682	0.567	0.413	0.654	0.181	0.296	0.899	0.935	0.647	0.752	0.920	0.898	0.434	0.489	0.418	0.447
preprandialKillingEffect_insecticideScalingFactor	0.133	0.300	0.097	0.029	0.300	0.093	0.096	0.107	0.300	0.042	0.094	0.300	0.121	0.300	0.086	0.081
preprandialKillingEffect_interactionFactor	-0.026	0.034	0.208	0.123	0.140	0.133	-0.058	-0.081	-0.131	-0.197	-0.029	-0.094	-0.020	-0.047	-0.187	-0.187
postprandialKillingEffect_baseFactor	0.067	0.067	0.014	0.014	0.066	0.066	0.028	0.019	0.144	0.144	0.026	0.033	0.000	0.000	0.101	0.101
postprandialKillingEffect_insecticideFactor	0.496	0.762	0.265	0.531	0.245	0.231	0.389	0.981	0.776	0.856	0.974	0.967	0.317	0.462	0.818	0.846
postprandialKillingEffect_insecticideScalingFactor	0.104	0.158	0.032	0.017	0.016	0.009	0.200	0.042	0.059	0.053	0.057	0.200	0.200	0.011	0.200	0.104

Legend: P2 = PermaNet 2.0, P3 = PermaNet 3.0, New B.= New Bussa, AG = *An. gambiae* s.s., AA = *An. arabiensis*.

References

1. Briët OJT, Hardy D, Smith TA: **Importance of factors determining the effective lifetime of a mass, long-lasting, insecticidal net distribution: a sensitivity analysis.** *Malar J* 2012, **11:20.** 20.

2. Maire N, Smith T, Ross A, Owusu-Agyei S, Dietz K, Molineaux L: **A model for natural immunity to asexual blood stages of *Plasmodium falciparum* malaria in endemic areas.** *Am J Trop Med Hyg* 2006, **75**(2 Suppl)**:** 19-31.

3. Tami A, Mubyazi G, Talbert A, Mshinda H, Duchon S, Lengeler C: **Evaluation of Olyset insecticide-treated nets distributed seven years previously in Tanzania.** *Malar J* 2004, **3**(1)**:** 19.

4. Kilian A, Byamukama W, Pigeon O, Gimnig J, Atieli F, Koekemoer LL, Protopopoff N: **Evidence for a useful life of more than three years for a polyester-based long-lasting insecticidal mosquito net in Western Uganda.** *Malar J* 2011, **10**(1)**:** 299.

5. Kilian A, Byamukama W, Pigeon O, Atieli F, Duchon S, Phan C: **Long-term field performance of a polyester-based long-lasting insecticidal mosquito net in rural Uganda.** *Malar J* 2008, **7:49.** 49.

6. Corbel V, Chabi J, Dabire RK, Etang J, Nwane P, Pigeon O, Akogbeto M, Hougard JM: **Field efficacy of a new mosaic long-lasting mosquito net (PermaNet® 3.0) against pyrethroid-resistant malaria vectors: a multi centre study in Western and Central Africa.** *Malar J* 2010, **9:113.** 113.

7. Briët OJT, Hardy D, Smith TA: **Importance of factors determining the effective lifetime of a mass, long-lasting, insecticidal net distribution: a sensitivity analysis.** *Malar J* 2012, **11**(1)**:** 20.

8. Irish S, N'Guessan R, Boko P, Metonnou C, Odjo A, Akogbeto M, Rowland M: **Loss of protection with insecticide-treated nets against pyrethroid-resistant *Culex quinquefasciatus* mosquitoes once nets become holed: an experimental hut study.** *Parasit Vectors* 2008, **1**(1)**:** 17.

9. Tediosi F, Maire N, Smith T, Hutton G, Utzinger J, Ross A, Tanner M: **An approach to model the costs and effects of case management of *Plasmodium falciparum* malaria in sub-saharan Africa.** *Am J Trop Med Hyg* 2006, **75**(2 Suppl)**:** 90-103.

10. N'Guessan R, Asidi A, Boko P, Odjo A, Akogbeto M, Pigeon O, Rowland M: **An experimental hut evaluation of PermaNet® 3.0, a deltamethrin-piperonyl butoxide combination net, against pyrethroid-resistant *Anopheles gambiae* and *Culex quinquefasciatus* mosquitoes in southern Benin.** *Trans R Soc Trop Med Hyg* 2010, **104**(12)**:** 758-765.

11. Djouaka RF, Bakare AA, Coulibaly ON, Akogbeto MC, Ranson H, Hemingway J, Strode C: **Expression of the cytochrome P450s, CYP6P3 and CYP6M2 are significantly elevated in multiple pyrethroid resistant populations of *Anopheles gambiae* s.s. from Southern Benin and Nigeria.** *BMC Genomics* 2008, **9:538.** 538.

12. Tungu P, Magesa S, Maxwell C, Malima R, Masue D, Sudi W, Myamba J, Pigeon O, Rowland M: **Evaluation of PermaNet 3.0 a deltamethrin-PBO combination net**

against *Anopheles gambiae* and pyrethroid resistant *Culex quinquefasciatus* mosquitoes: an experimental hut trial in Tanzania. *Malar J* 2010, **9:21.** 21.

13. Kitau J, Oxborough RM, Tungu PK, Matowo J, Malima RC, Magesa SM, Bruce J, Mosha FW, Rowland MW: **Species shifts in the *Anopheles gambiae* complex: Do LLINs successfully control *Anopheles arabiensis*?** *PLoS ONE* 2012, **7**(3)**:** e31481.

14. Adeogun AO, Olojede JB, Oduola AO, Awolola TS: **Efficacy of a combination long lasting insecticidal net (PermaNet® 3.0) against pyrethroid resistant *Anopheles gambiae* s.s. and *Culex quinquefasciatus*: an experimental hut trial in Nigeria.** *Nigerian Journal of Clinical and Biomedical Research* 2012, **6**(1)**:** 37-50.

15. Koudou BG, Koffi AA, Malone D, Hemingway J: **Efficacy of PermaNet® 2.0 and PermaNet® 3.0 against insecticide-resistant *Anopheles gambiae* in experimental huts in Cote d'Ivoire.** *Malar J* 2011, **10:172.** 172.

16. Chandre F, Darrier F, Manga L, Akogbeto M, Faye O, Mouchet J, Guillet P: **Status of pyrethroid resistance in *Anopheles gambiae* sensu lato.** *Bull World Health Organ* 1999, **77**(3)**:** 230-234.

17. Lines JD, Curtis CF, Myamba J, Njau R: **Tests of repellent or insecticide impregnated curtains, bednets and anklets against malaria vectors in Tanzania.**World Health Organization; 1985, **WHO/VBC/85.920**.

18. Lines JD, Myamba J, Curtis CF: **Experimental hut trials of permethrin-impregnated mosquito nets and eave curtains against malaria vectors in Tanzania.** *Med Vet Entomol* 1987, **1**(1)**:** 37-51.

19. Van Bortel W, Chinh VD, Berkvens D, Speybroeck N, Trung HD, Coosemans M: **Impact of insecticide-treated nets on wild pyrethroid resistant *Anopheles epiroticus* population from southern Vietnam tested in experimental huts.** *Malar J* 2009, **8:248.** 248.

20. World Health Organisation: **Guidelines for testing mosquito adulticides for indoor residual spraying and treatment of mosquito nets.** Geneva: World Health Organisation; 2006, **WHO/CDS/NTD/WHOPES/GCDPP/2006.3**.
