# Supplementary material for: Effects of pyrethroid resistance on the cost effectiveness of a mass distribution of long-lasting insecticidal nets: a modelling study
Source: Malar J. 2013 Feb 25;12:77. doi: 10.1186/1475-2875-12-77 (PMC3598792; doi:10.1186/1475-2875-12-77)
Supplement: Additional file 3 — Health economics. [file 1475-2875-12-77-S3.pdf]

Additional file 3 Health economics

Disability adjusted life years (DALYs)
DALYs are the sum of years of life lost (YLL) and years lived with disability adjusted for the severity of the disability (YLDs), due to ill health. DALYs were calculated using the formulae and standard described by Murray and Lopez [1], with a 3% discount rate. For age specific disability weights for uncomplicated malaria episodes, values proposed by Murray and Lopez [1] for malaria episodes were taken, which were 0.211 for zero to four year olds, 0.195 for five to 14 year olds, and 0.172 for ages 15 and older. Murray and Lopez [1] do not distinguish between uncomplicated and severe episodes. For severe episodes, a weight of 0.6 was chosen.

For untreated neurological sequelae, a weight of 0.473 was used for all ages [1], whereas for treated sequelae, slightly lower, age dependent values of 0.436 for zero to four year olds, and 0.435 for five year olds and over were used [1]. Mild anaemia, which has low weights of 0.013 for 45–59 year olds and 0.012 for the other ages, both treated and untreated [1], is not a standard output of the OpenMalaria models, and was ignored.

Following a study by Tediosi and colleagues [2], age specific life expectancies were based on the life table from Butajira, Ethiopia [3]. The duration of sequelae were measured from the age of the event until the life expectancy. Similarly, the YLLs were calculated from the age at death until the life expectancy. The duration of severe untreated episodes was taken as 17.5 days (the average of two to three weeks [4]). The duration of severe treated episodes was taken as 8.75 days (the average of 6.5 days for cerebral malaria and 11 days for severe anaemia [5]). For the duration of untreated uncomplicated malaria episodes, another study used two weeks [6], but did not quote empirical evidence. For the duration of treated uncomplicated malaria episodes, Snow and colleagues used 5.1 days for patients in the zero to 14 year age range [5]. For uncomplicated treated and untreated episodes, data from malaria therapy patients [7] were re-analysed using a modelling approach, using health system settings as used in the simulation experiments used for this study, with an overall treatment probability of 5% per five-day time step if this time step contains one or more days with a recorded fever, and a maximum episode length of 30 days (a recorded fever 31 days after an initial malaria fever is attributed to a new episode). This five-day treatment probability of 5% corresponds to a 2.5% daily treatment probability of a recorded fever. With this daily treatment probability, the duration of a treated episode was 4.32 days, and of an untreated episode was 5.02 days. This corresponds very well with the value of 5.1 days used by Snow and colleagues. Durations for malaria episodes are summarized in the Table. YLLs were based on the number in each age group of direct (*nDirDeaths*) and indirect malaria deaths (*nIndDeaths*). YLDs were based on the number in each age group of:

- recoveries from untreated malaria episodes with sequelae outside of hospitals, which was the number of recoveries from malaria episodes with sequelae (*nSeq*) minus the number of recoveries in hospital from malaria episodes with sequelae (*nHospitalSeqs*);
- recoveries from treated malaria episodes with sequelae (*nHospitalSeqs*);
- recoveries from severe treated malaria (in hospital) without sequelae (*nHospitalRecovs*);
- recoveries from severe untreated malaria episodes, which was the number of severe episodes (*nSevere*) minus the number of direct malaria deaths (*nDirDeaths*), minus the number of inpatient treatments (*nTreatments3*), plus the number of deaths in hospital (*nHospitalDeaths*), minus the number of recoveries from malaria episodes with sequelae (*nSeq*), plus the number of recoveries in hospital from malaria episodes with sequelae (*nHospitalSeqs*);

- recoveries from treated uncomplicated episodes, which was the number of first (*nTreatments1*) and second line treatments (*nTreatments2*), assuming that second line treatment is still for uncomplicated cases, and that the duration of the episode is counted twice due to recrudescence (in OpenMalaria, a second line treatment is only given to an old episode within the period of the health system memory (set to 30 days in these scenarios), as thereafter, it would be counted as a new episode);
- recoveries from untreated uncomplicated episodes, which was the number of uncomplicated episodes (*nUncomp*), minus the number of first line treatment (*nTreatments1*). The number of second line treatment (*nTreatments2*) was not subtracted from (*nUncomp*), because these were already counted in the first line treatment number.

The weight of the mayor components of DALYs for malaria illness and death in non-intervention scenarios is illustrated depending on EIR (Figure 1) and model variant (Figure 2).

Table. Duration of malaria episodes used in DALY calculation

Episode	Duration (years)
Uncomplicated episodes treated	0.01320548
Uncomplicated episodes untreated	0.01375342
Severe episodes treated	0.02397260
Severe episodes untreated	0.04794521

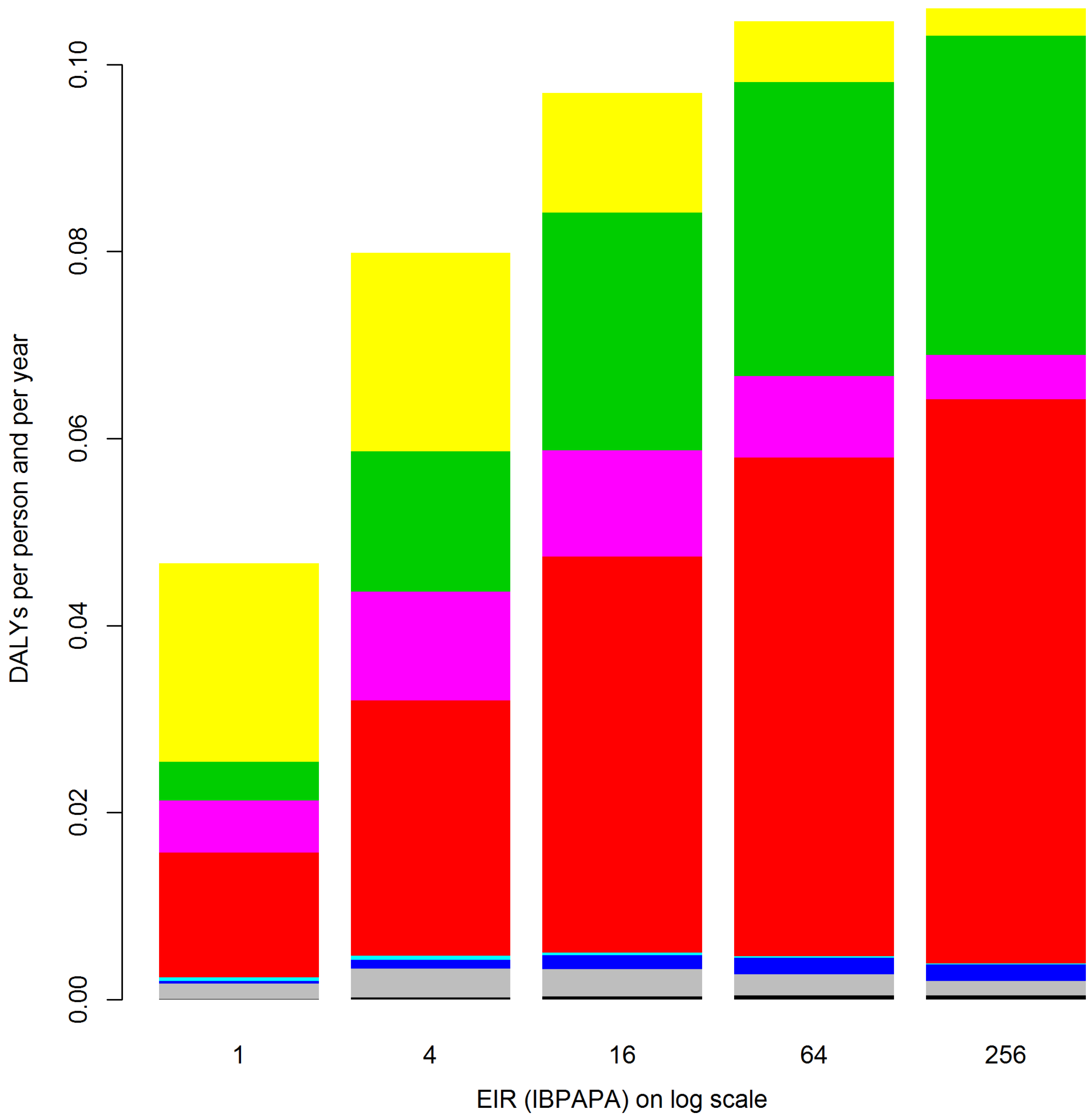

Figure 1. DALYs due to malaria in non-intervention scenarios in the base model variant (R0000). Each bar element shows the number of disability adjusted life years (DALYs) due to malaria related illness or death in the base model (R0000), averaged over of 10 simulation runs (each with unique random seed). Models were run without intervention other than the reference health system [2] with artemisinin combination therapy [8] with a population size of 100,000. Bar elements are colour coded as follows: black: uncomplicated episodes in under five year olds (U5), grey: uncomplicated episodes in over five year olds (O5), dark blue: severe episodes and sequelae in U5, light blue: severe episodes and sequelae in O5, red: indirect malaria deaths in U5, magenta: indirect malaria deaths in O5, green: direct deaths in U5, yellow: direct deaths in O5.

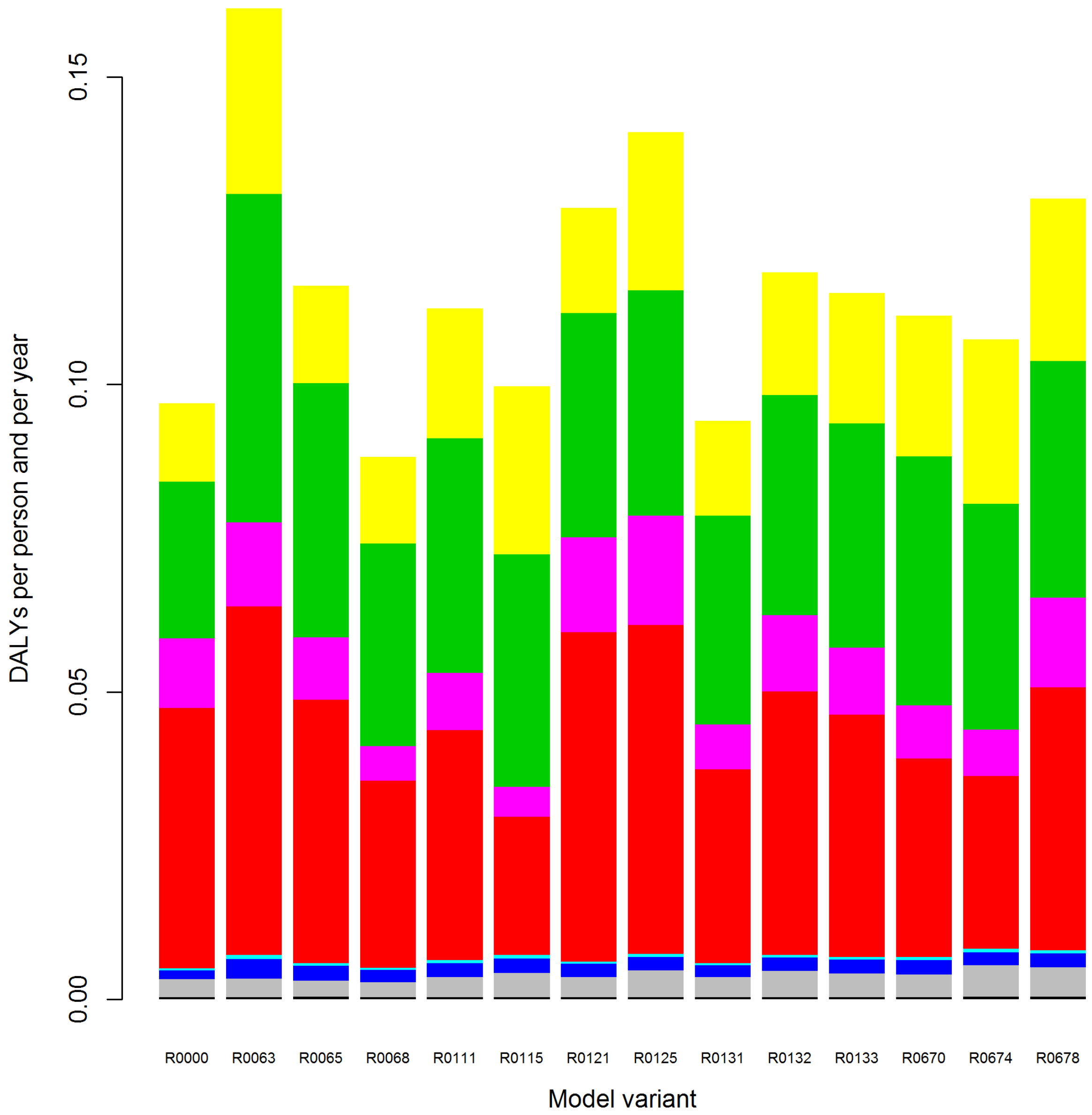

Figure 2. DALYs due to malaria in non-intervention scenarios at a transmission level of 16 IBPAPA. Each bar element shows the number of disability adjusted life years (DALYs) due to malaria related illness or death at a transmission level of 16 infectious bites per person and per annum (IBPAPA) depending on the model variant, averaged over of 10 simulation runs (each with unique random seed). Bar elements are colour coded as in Figure 1.

Health systems costs
The health system component of the scenarios corresponded to the reference scenario described by Tediosi and colleagues [2], with the difference that artemisinin combination therapy (ACT) was used as first and second line treatment for uncomplicated episodes with a cure rate of 85% [8]. Costs were calculated as previously reported [8] based on the number of first (*nTreatments1*) and second line treatments (*nTreatments2*), deaths in hospital (*nHospitalDeaths*), recoveries in hospital with (*nHospitalSeqs*) and without sequelae (*nHospitalRecovs*), and inflation adjusted to 2012 US$.

Costing of nets
Vestergaard-Frandsen provided the following 2012 unit prices (port delivery) of $3.25 US$ for P2 and US$4.90 for P3.

According to Mueller and colleagues [9], the extra campaign cost per net in 2004 US$1.62, which, with an inflation correction of 120% [10] corresponds to 2012US$1.94. Thus, campaign costs per net would be 3.25+1.94=5.19 and 4.90+1.94= 6.84 for P2 and P3, respectively. These cost estimates are subject to market dynamics, order size, etc., and are indicative only.

Net health benefit (NHB)
In order to convert costs to DALYs, a 1993 ceiling ratio of US$150 [11,12] was used. This was converted [10] in to 2012US$235.28. Thus, 1 US$ = 1/235.28 DALY.

References

1. Murray CJL, Lopez AD: *The global burden of disease: a comprehensive assessment of mortality and disability from diseases, injuries, and risk factors in 1990 and projected to 2020*. Harvard: Harvard University Press; 1996.

2. Tediosi F, Maire N, Smith T, Hutton G, Utzinger J, Ross A, Tanner M: **An approach to model the costs and effects of case management of *Plasmodium falciparum* malaria in sub-saharan Africa.** *Am J Trop Med Hyg* 2006, **75**(2 Suppl)**:** 90-103.

3. INDEPTH Network: *Model Life Tables for Sub-Saharan Africa*. Aldershot, England: Ashgate; 2004.

4. John DT, Petri WA, Markell EK, Voge M: *Markell And Voge's Medical Parasitology*, 9 edn. Elsevier Health Sciences; 2006.

5. Snow R, Craig M, Newton C, Steketee RW: **The public health burden of *Plasmodium falciparum* malaria in Africa: Deriving the numbers.**Bethesda, Maryland.: Fogarty International Center, National Institutes of Health.; 2003.

6. Mueller DH, Wiseman V, Bakusa D, Morgah K, Dare A, Tchamdja P: **Cost-effectiveness analysis of insecticide-treated net distribution as part of the Togo Integrated Child Health Campaign.** *Malar J* 2008, **7:73.** 73.

7. Collins WE, Jeffery GM: **A retrospective examination of the patterns of recrudescence in patients infected with *Plasmodium falciparum*.** *Am J Trop Med Hyg* 1999, **61**(1 Suppl)**:** 44-48.

8. Tediosi F, Maire N, Penny M, Studer A, Smith TA: **Simulation of the cost-effectiveness of malaria vaccines.** *Malar J* 2009, **8:127.** 127.

9. Mueller DH, Wiseman V, Bakusa D, Morgah K, Dare A, Tchamdja P: **Economic evaluation of the Togo Integrated Child Health Campaign 2004.**London: 2007.

10. US Inflation Calculator. 2012.

11. The World Bank: **World development report 1993: Investing in health.**World Bank and Oxford University Press; 1993.

12. Maire N., Shillcutt S, Walker DG, Tediosi F, Smith T: **Cost effectiveness of the introduction of a pre-erythrocytic malaria vaccine into the Expanded Program on Immunization in sub-Saharan Africa: analysis of uncertainties using a stochastic individual-based simulation model of *Plasmodium falciparum* malaria.** *Value Health* 2011, **14** 1028-1038.
