# Supplementary material for: Effects of pyrethroid resistance on the cost effectiveness of a mass distribution of long-lasting insecticidal nets: a modelling study
Source: Malar J. 2013 Feb 25;12:77. doi: 10.1186/1475-2875-12-77 (PMC3598792; doi:10.1186/1475-2875-12-77)
Supplement: Additional file 4 — Effectiveness of a mass distribution of PermaNet 2.0 bed nets depending on insecticide resistance status, compared to a susceptible population; Legend: See Figure 4. [file 1475-2875-12-77-S4.pdf]

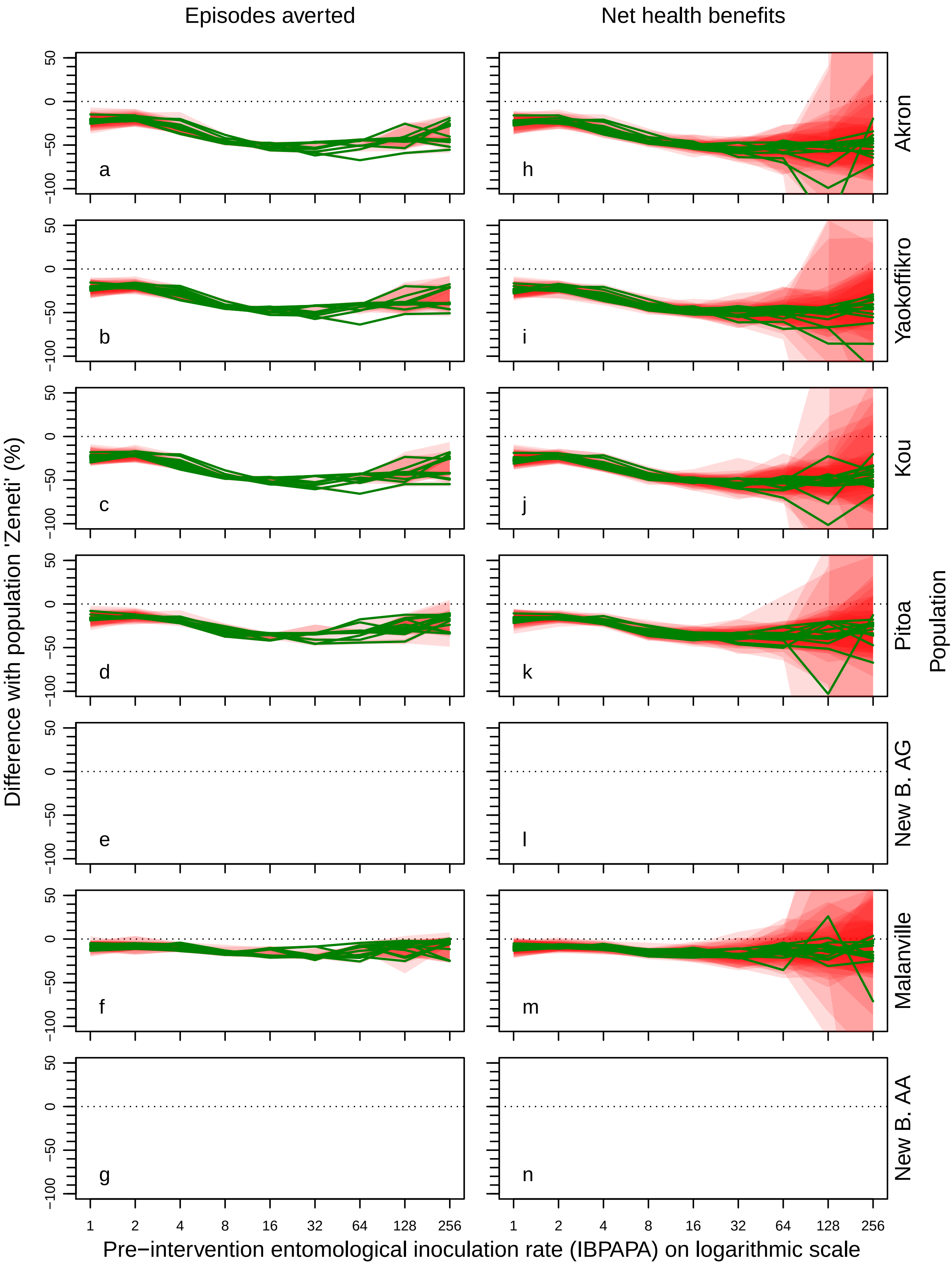

Additional file 4. Effectiveness of a mass distribution of PermaNet 2.0 bed nets depending on insecticide resistance status, compared to a susceptible population. Legend: See Figure 4.
